# Apoptotic vesicles derived from bone marrow mesenchymal stem cells increase angiogenesis in a hind limb ischemia model via the NAMPT/SIRT1/FOXO1 axis

**DOI:** 10.1186/s13287-025-04245-1

**Published:** 2025-03-01

**Authors:** Jinxing Chen, Zekun Shen, Bingyi Chen, Shuang Liu, Yifan Mei, Kai Li, Ziyang Peng, Chaoshuai Feng, Weiyi Wang, Shaoying Lu

**Affiliations:** 1 Department of Vascular Surgery, The First Affiliated Hospital of Xi’an JiaoTong University, Xi’an, Shaanxi 710061 P.R. China; 2 Department of Otorhinolaryngology-Head and Neck Surgery, The First Affiliated Hospital of Xi’an Jiaotong University, Xi’an, Shaanxi 710061 P.R. China; 3 School of Future Technology, National Local Joint Engineering Research Center for Precision Surgery & Regenerative Medicine, Xi’an Jiaotong University, Xi’an, Shaanxi 710061 China; 4 Department of Spine Surgery, Hong Hui Hospital, Xi’an Jiaotong University, 555 You Yi Dong Road, Xi’an, Shaanxi 710054 P.R. China

**Keywords:** Apoptotic vesicles, Mesenchymal stem cell, Angiogenesis, Human umbilical vein endothelial cells, Hind limb ischemia model

## Abstract

**Background:**

Chronic limb-threatening ischemia (CLTI) is the most severe form of peripheral arterial disease (PAD). Mesenchymal stem cell (MSC) transplantation holds promise as a treatment for CLTI; however, the harsh local environment poses challenges to its effectiveness. Apoptotic vesicles (ApoVs) are extracellular vesicles produced by cells undergoing apoptosis, and they can carry various biomolecules from their parent cells, including proteins, RNA, DNA, lipids, ions, and gas neurotransmitters. ApoVs play significant roles in anti-inflammatory responses, anti-tumor activities, and tissue regeneration through intercellular communication, and they have demonstrated potential as drug carriers. In this study, we investigated the potential of bone marrow stem cell (BMSC)-derived ApoVs for treating CLTI.

**Methods:**

In vivo, we explored the therapeutic effect of ApoVs on a hindlimb ischemia model through Laser Doppler, matrigel plug assay, and histological analysis. In vitro, we analyzed the effects of ApoVs on the proliferation, migration, and angiogenesis of HUVECs and explored the uptake process of ApoVs. In addition, Proteomic analysis, western blotting, quantitative real-time PCR, shRNA, and siRNA were used to analyze ApoVs-induced HUVECs activation and downstream signaling pathways.

**Results:**

BMSCs transplantation showed improvement in a hind limb ischemia model, and this effect still exists after apoptosis of BMSCs. Subsequently, ApoVs of BMSCs were isolated and found to improve mouse hind limb ischemia in vivo. In vitro, ApoVs can be ingested by HUVECs through dynamin-, clathrin-, and caveolin-mediated endocytosis and promote its proliferation, migration, and angiogenesis. Mechanistically, ApoVs transferred NAMPT to HUVECs, therefore activating the NAMPT/SIRT1/FOXO1 axis, influencing the transcriptional activity of FOXO1, and promoting angiogenesis.

**Conclusions:**

Our results demonstrate that the transplanted BMSCs can ameliorate hindlimb ischemia by releasing ApoVs during apoptosis. The main mechanism of this effect is promoting the proliferation, migration, and angiogenesis of HUVECs through the NAMPT/SIRT1/FOXO1 axis. This study provides different insights into the therapeutic mechanisms through BMSCs and suggests a promising direction for ApoVs transplantation.

**Clinical trial number:**

Not applicable.

**Graphical Abstract:**

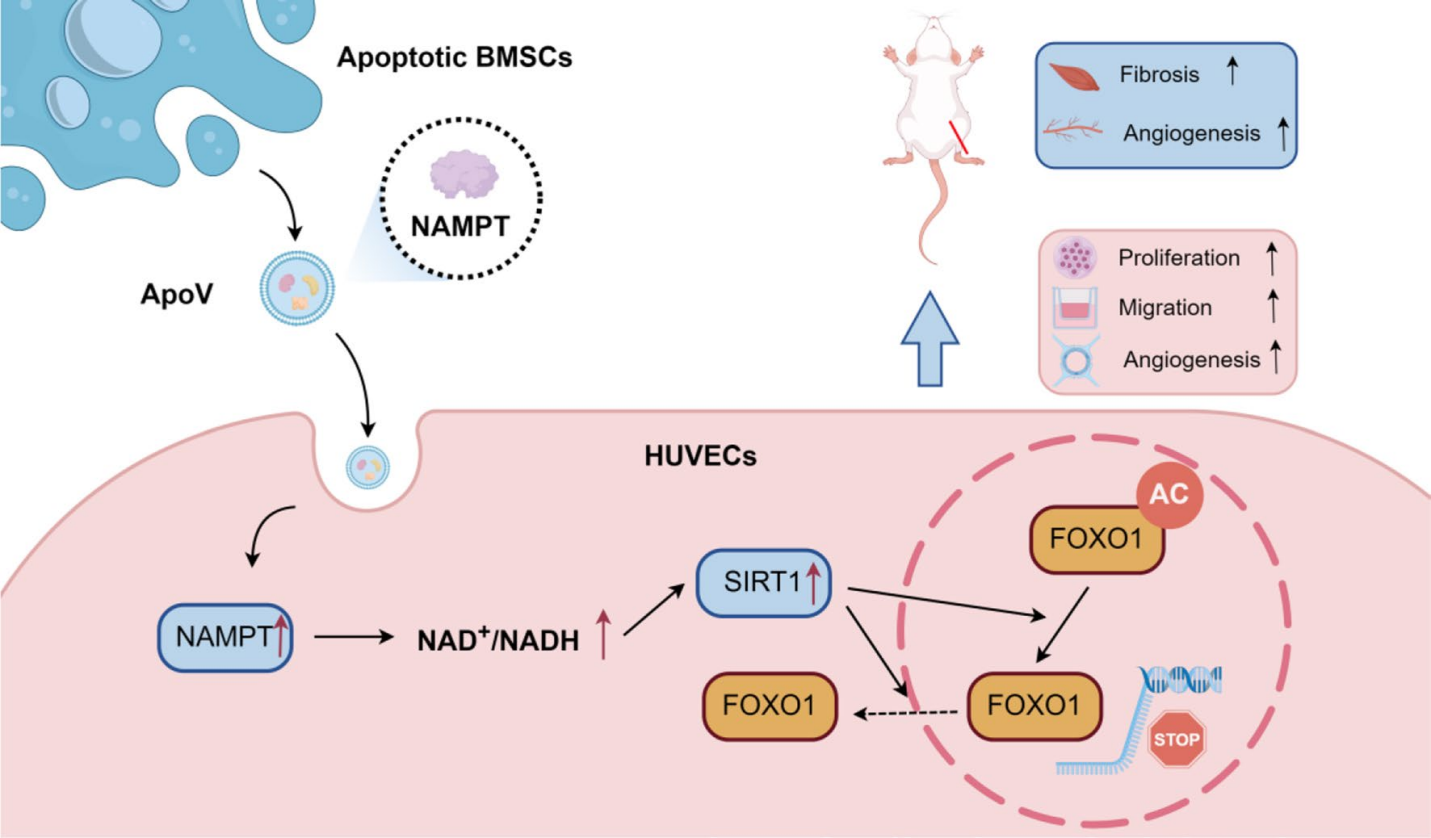

**Supplementary Information:**

The online version contains supplementary material available at 10.1186/s13287-025-04245-1.

## Background

Peripheral arterial disease (PAD) is a chronic vascular condition caused by atherosclerosis affecting arteries outside the e heart and brain. The most severe manifestation of PAD is chronic limb-threatening ischemia (CLTI), characterized by rest pain, tissue loss, and a heightened risk of amputation or death [1]. Atherosclerotic stenosis and occlusion in the lower extremity arteries disrupt circulation and lead to significant changes in leg tissues [2]. Repeated ischemic episodes result in severe muscle damage, fibrosis, and mitochondrial dysfunction in skeletal muscle cells [3]. Furthermore, ischemia-induced microvascular dysfunction causes endothelial damage in CLTI patients [4] substantially increasing their risk of amputation [5, 6]. Although surgical and endovascular revascularization are effective treatments for CLTI, approximately 20% of patients are ineligible for these procedures. As a result, stimulating angiogenesis to enhance tissue perfusion has emerged as a promising therapeutic strategy for this subset of patients.

Due to their ability to induce angiogenesis, mesenchymal stem cells (MSCs) have been extensively utilized for tissue repair and the treatment of various ischemic diseases [7]. Initially, researchers believed that MSCs’ therapeutic effect was attributed to their migration in target tissues and homing ability after transplantation. However, subsequent studies revealed that MSCs’ regeneration effect was mainly due to the secretion of bioactive factors. These factors facilitate intercellular communication and regulate crucial processes such as cell proliferation, differentiation, and anti-inflammatory responses [8–10]. Research has demonstrated that MSCs significantly enhance their release of chemokines and angiogenic factors under hypoxic conditions [11, 12] Moreover, inflammation-activated MSCs can attract immune cells, inducing the production of nitric oxide synthase (iNOS) [13]. In addition, MSCs-conditioned medium can promote the function of endothelial cells and recruit macrophages to wound sites [14]. However, an ischemic and hypoxic environment can lead to insufficient survival of transplanted MSCs, which is unfavorable for revascularization or tissue recovery. The poor survival rate of MSCs may be related to mechanical leakage of cells, necrosis and apoptosis, inflammation, oxidative stress, and extracellular matrix disorder [15]. Therefore, improving the survival rate of MSCs or exploring alternative therapeutic strategies has become a key area of interest for researchers.

During apoptosis, cells release various substances, including apoptotic vesicles (ApoVs), which contribute to intercellular communication and play critical roles in development, immunity, and tissue homeostasis. For example, T cell-derived ApoVs hydrolyzed cGAMP to alleviate radiation enteritis [16]. 3D-cultured adipose MSC-ApoVs were more effective in promoting ischaemic skin flap survival [17]. Additionally, ApoVs can inherit DNA, RNA, proteins, lipids, and organelles from apoptotic cells for signal transduction. It has been reported that ApoVs inherit proteins and microRNAs (miRNAs) from MSCs and facilitate angiogenesis by transferring these molecules to endothelial cells [17–19]. Despite these promising findings, research into ApoVs remains relatively limited, and their underlying biological mechanisms are unclear. Further investigation is needed to uncover their potential therapeutic applications and the pathways through which they exert their effects.

In this study, we found that bone marrow mesenchymal stem cells (BMSCs) transplanted into the ischemic hindlimbs retained their therapeutic potential even after undergoing apoptosis, which may be related to the release of ApoVs. To verify the therapeutic effect of ApoVs, we induced BMSCs apoptosis and isolated ApoVs in vitro. Next, we verified the therapeutic effect of ApoVs in the hindlimb ischemia model and assessed their influence on the proliferation, migration, and angiogenic capabilities of human umbilical vein endothelial cells (HUVECs). Mechanistically, we found that ApoVs transferred nicotinamide phosphoribosyl transferase (NAMPT) to HUVECs, activating the NAMPT/SIRT1/FOXO1 signaling axis. In summary, our data indicate that ApoVs from BMSCs promote the function of HUVECs, providing new insights for the treatment of CLTI.

## Materials and methods

### Animal models

The work has been reported in line with the ARRIVE guidelines 2.0. Male BALB/c mice aged 6–8 weeks were purchased from the Laboratory Animal Center of Xi’an Jiaotong University. The mice were housed in a constant temperature and suitable humidity (22 °C, 40–60% humidity) environment with a12h light/dark cycle. For the hind limb ischemia model, anaesthetizing mice with 1.5% isoflurane, bilateral ligation of the left femoral artery in the mice was performed to induce hind limb ischemia. Use a heating pad during the surgery to ensure the temperature of the mice. For clodronate treatment, we intraperitoneally injected 200 µL/20 g of clodronate liposomes (#SN-ML-E005, SunLipo NanoTech, Shanghai, China) the day before hind limb ischemia. Then, we injected the liposomes twice a week to maintain macrophage clearance. PBS-containing liposomes were used as controls. For BMSCs or ApoVs transplantation, 1 × 10^6^ BMSCs or 100 µg ApoVs in 100 µL of PBS were intramuscularly injected into the gastrocnemius muscle of the ischemic limb, with the same volume of PBS used as the control. Mice were divided into six groups: hind limb ischemia mice with PBS (PBS); hind limb ischemia mice with BMSCs (BMSCs); hind limb ischemia mice with PBS and control liposomes (PBS + Ctrl-lip); hind limb ischemia mice with PBS and clodronate liposomes (PBS + Clo-lip); hind limb ischemia mice with ApoVs and control liposomes (ApoVs + Ctrl-lip); hind limb ischemia mice with ApoVs and clodronate liposomes (ApoVs + Clo-lip). All animals were randomly grouped by computer. Briefly, the computer assigned each mouse a random number and divided it by the number of groups to obtain the remainder for grouping. Each group used 6 mice and researchers were not involved in the allocation process of mice.

### Histological and Immunofluorescence staining

Animals were euthanized with intraperitoneal injection of excessive pentobarbital (500 µL). After euthanasia, tissue samples of the gastrocnemius muscle taken at 7, 14, and 28 days were paraffin embedded. Hematoxylin and eosin (H&E) staining and Masson’s trichrome staining (Solarbio, Beijing, China) were performed on 6 μm thick slices. For immunofluorescence staining, optimal cutting temperature (OCT) was used to embed the tissues. The frozen Sect. (7 μm) were blocked in 5% BSA at room temperature for 1 h and then incubated with a CD31 antibody (1:500, #ab76533, Abcam) at 4 °C for 12 h. Subsequently, the sections were incubated with a Cy3-labeled secondary antibody (#A0516, Beyotime) at room temperature for 1 h, followed by staining with 4’-6-diamidino-2-phenylindole (DAPI, #C1002, Beyotime). For BMSCs apoptosis, the cells were incubated with an anti-cleaved caspase 3 antibody (1:250, # ab32042, Abcam) at 4 °C for 12 h and then stained with DAPI. A fluorescence microscope (Lecia, Wetzlar, Germany) was used for image acquisition, and 3 fields of view were randomly selected and quantified via ImageJ (2.3.0).

### Bioluminescence imaging (BLI)

BLI was performed via an IVIS imaging system (Xenogen Corporation, Hopkinton, MA, USA). Briefly, the survival of BMSCs^Fluc^ in mice on Days 1, 3, 5, and 7 after transplantation was evaluated. After the mice were anesthetized with isoflurane, D-luciferin (150 mg/kg, Beyotime) was injected intraperitoneally, and the signal intensity was measured via the IVIS system. The average intensity of the region of interest (ROI) represents the survival status of the cells.

### Laser doppler perfusion

Scanning of ischemic limb perfusion in mice was performed via a laser Doppler perfusion imager (Moor Instruments, Devon, United Kingdom) after isoflurane anesthesia. Blood flow monitoring of the ischemic limbs was performed on Days 1, 3, 7, 14, and 21 after the operation. The left-to-right ratio (ischemia/normal) was used to represent the recovery of blood flow in the hind limbs of each mouse.

### Matrigel plug assay

To measure the in vivo angiogenic capacity of ApoVs, we performed a Matrigel plug assay according to previously reported method [20]. Briefly, 6–8 weeks old BALB/c mice (with clo-lip or ctrl-lip treatment) were anesthetized with isoflurane, 200 µg ApoVs were suspended in 100 µL PBS and mixed with 450 µL Matrigel (#082704, ABW) with the same volume of PBS used as the control. Matrigel plugs were transplanted subcutaneously into the abdomens of mice. The mice were euthanized at 14 days, and the Matrigel plugs were collected for H&E and CD31 immunofluorescence staining. The experiment was repeated three times.

### Cell culture

Human BMSCs were purchased from Procell (#CP-H166, Procell), and the cells were cultured in DMEM/F12 supplemented with 10% fetal bovine serum (FBS) (#164210, Procell) and 1% penicillin‒streptomycin (#C100C5, NCM Biotech). HUVECs were purchased from the American Type Culture Collection (ATCC). The medium used for HUVECs was endothelial cell medium (ECM; ScienCell) containing 10% FBS supplemented with endothelial cell growth supplement (ScienCell). The cells were incubated in an incubator humidified with 5% CO_2_ at 37 °C. The cells used in this study were within 6 passages.

### Flow cytometry (FCM)

For the characterization of BMSCs, the specific steps can be found in our previous research [20]. Briefly, BMSCs were stained with fluorescence-labeled monoclonal antibodies. A flow cytometer (NovoCyte, Agilent, USA) was used to analyze the stained cells. NovoExpress (1.6.2) software was used to analyze the data. The antibodies used in this study were as follows: CD29-FITC (#11–0299–41, eBioscience, San Diego, CA, USA), CD44-PE (#12–0441–81, eBioscience), CD90-FITC (#11–0909–41, eBioscience), CD105-APC (#17–1057–41, eBioscience), CD34-APC (#17–0349–41, eBioscience), CD45-PE (#12–0456–41, eBioscience).

### Isolation and characterization of ApoVs

ApoVs were isolated from the cell supernatant of BMSCs. First, BMSCs were treated with 0.5 µM staurosporine (STS) (#HY-15141, MCE) for 12 h to induce apoptosis. The supernatant was subsequently collected and centrifuged at 300 × g for 10 min to remove cell debris. After the supernatant was collected, centrifugation was continued at 3000 × g for 30 min. Finally, the supernatant was collected and centrifuged at 16,000 × g for 1 h, after which the pellet was resuspended in sterile PBS. The morphology of the obtained ApoVs was observed via transmission electron microscopy (TEM, Hitachi), and the particle diameter was measured via nanoparticle tracking analysis (NTA). The expression of cleaved caspase 3 (1:500, #ab32042, Abcam) and caspase 3 (1:5000, #ab32351, Abcam) was determined via Western blotting.

### Proliferation and apoptosis analysis

A Cell Counting Kit-8 (CCK-8, #C0005, TargetMol) assay was used to analyze cell proliferation and apoptosis following the manufacturer’s protocol. For proliferation analysis, HUVECs (5 × 10^3^ cells/well) were seeded in 96-well plates and incubated with different concentrations of ApoVs for 24 h and 48 h. Then, the medium in each well was replaced with 10% CCK-8 basal medium, and the mixture was incubated at 37 °C for 2 h. The proliferation rate was calculated after the absorbance was measured with a microplate reader (Bio-Rad, Hercules) at 450 nm. For apoptosis analysis, BMSCs (8 × 10^3^ cells/well) were seeded in 96-well plates and incubated with different concentrations of STS for 12 h. The apoptosis rate was calculated by measuring the absorbance at 450 nm 2 h after the addition of CCK-8.

Cell apoptosis was assessed by flow cytometry via an apoptosis detection kit (#559763, BD Biosciences) containing Annexin V-PE/7-AAD. Briefly, BMSCs (10 × 10^4^ cells/well) were seeded in 6-well plates and digested after the addition of 0.5 µM STS for 12 h. Annexin V and 7-AAD were stained according to the manufacturer’s protocol and subsequently tested via FCM.

### ApoVs uptake by HUVECs in vitro

Fluorescence staining was used to observe the internalization of ApoVs by HUVECs. Isolated ApoVs were labeled with Dil (#C1036, Beyotime). ApoVs were incubated with Dil for 10 min at room temperature and then centrifuged at 16,000 × g and 4 °C for 1 h. Labeled ApoVs were resuspended in complete medium, and the concentration was adjusted to 20 µg/mL. After incubation with the Dil-ApoVs for 24 h, the HUVECs were washed 3 times with PBS and then fixed in 4% paraformaldehyde for 20 min. After the HUVECs were washed 3 times with PBS, the cytoskeleton and nucleus of the HUVECs were stained with anti-Tracker Green-488 (#C2201S, Beyotime) and DAPI.

For the FCM assay, HUVECs (10 × 10^4^ cells/well) were seeded in 24-well plates, and ApoVs (20 µg/mL) labeled with DiO (#C1038, Beyotime) were added for 1, 6, 12, or 24 h. The rate of ApoVs uptake by HUVECs was determined by detecting DiO-positive cells in the HUVECs.

For ApoVs uptake inhibitors, HVUECs were first incubated in complete medium containing dynasore (#HY-15304, MCE, 12.5–100 µM), chlorpromazine (#HY-12708, MCE, 6.25-50 µM), Pitstop 2 (#HY-115604, MCE, 12.5–100 µM), nystatin (NYST, #HY-17409, MCE, 10–80 µM), ethylisopropylamiloride (EIPA, #HY-101840, MCE, 12.5–100 µM), methyl-β-cyclodextrin (MβCD, #HY-101461, MCE, 0.1–100 µg/mL) or heparin (#HY-17567, MCE, 0.1–100 µg/mL) at 37 °C for 30 min, and then, DiO-ApoVs (20 µg/mL) were added for 6 h. The percentage of DIO-positive HUVECs was detected via FCM. The experiment was repeated three times.

### Wound healing assay

HUVECs were seeded in 6-well plates, and scratch wounds were made on the cells via sterile 200 µL micropipettes when the cells reached 90% confluence. Different concentrations of ApoVs were resuspended in serum-free medium, the samples were added to a 6-well plate and incubated for 12 h, and photographs were taken. Five fields were randomly selected for each well. The results were analyzed via ImageJ (2.3.0).

### Transwell migration assay

HUVECs (8 × 10^3^) resuspended in 100 µL of serum-free medium were added to the upper chamber (8 μm, Corning), and medium containing ApoVs and 10% serum was added to the lower chamber. After 24 h, the chamber was washed 3 times with PBS and fixed with 4% paraformaldehyde for 15 min, followed by 0.1% crystal violet staining for 15 min. Three randomly selected visual fields were recorded under an inverted microscope (Olympus). The experiment was repeated 3 times.

### Tube formation assay

Fifty microliters of Matrigel (#082704, ABW) were plated in a 96-well plate and incubated at 37 °C for 30 min until the Matrigel solidified. HUVECs (1.3 × 10^4^ cells/well) were resuspended in serum-free medium containing different concentrations of ApoVs and seeded in 96-well plates precoated with Matrigel for 4 h. Photographs were taken via an inverted microscope (Olympus), and the number of nodes/junctions under the field of view was counted.

### qRT‒PCR

Total RNA was extracted with TRIzol agent (#15596026CN, Invitrogen) according to the manufacturer’s protocol. mRNA was reverse transcribed to cDNA with SweScript All-in-One RT SuperMix for qPCR (#G3337-50, Servicebio). qRT‒PCR was performed on a CFX96 Real-Time PCR system (Bio-Rad, USA) with RealStar Fast SYBR qPCR Mix (#A301, GenStar), and the relative expression levels of the mRNAs were calculated. The gene expression level was calculated according to the 2^−ΔΔCt^ method and normalized to GAPDH. The primers used in this study are shown in Table S1.

### Proteomic analysis

Protein lysates of HUVECs and HUVECs-ApoVs were prepared separately. Protein concentration determination and SDS‒PAGE were performed first, followed by tandem mass tag (TMT) peptide labeling. LC‒MS/MS analysis was performed via a Q Exactive HF instrument (Thermo Fisher, USA). Finally, the identified differentially expressed proteins were analyzed via the Gene Ontology (GO) and Kyoto Encyclopedia of Genes and Genomes (KEGG) databases for bioinformatic analysis.

### NAD+/NADH analysis

NAD^+^/NADH was assessed with an NAD^+^/NADH assay kit (#S0175, Beyotime) according to the manufacturer’s instructions. The absorbance was measured at 450 nm using a microplate reader. NAD_total_ and NADH were calculated separately in combination with the standard curve. NAD^+^/NADH is equal to [NAD_total_ - NADH]/NADH. The experiment was repeated three times.

### Generation of stable cell lines

The shRNA sequence of NAMPT was cloned and inserted into the pHBLV-U6-MCS-PGK-PURO plasmid (HANBIO) to silence NAMPT in human BMSCs. After 24 h of infection of BMSCs with lentivirus according to the manufacturer’s instructions, the BMSCs were screened with 2 µg/mL puromycin (#ST551, Beyotime). The effectiveness of shRNA silencing was confirmed by Western blotting. BMSCs stably transfected with sh-NC or sh-NAMPT were prepared, and ApoVs were separated and extracted for subsequent study. The sh-NC and sh-NAMPT sequences are shown in Table S2.

### siRNA-mediated knockdown

The siRNA negative control (si-NC) and siRNA- sirtuin 1 (si-SIRT1, HANBIO, China) were transfected into HUVECs via Lipo8000 transfection reagent (#C0533, Beyotime) according to the manufacturer’s protocol. The transfection efficacy was verified after 72 h by Western blotting.The si-NC and si-SIRT1 sequences are shown in Table S2.

### Western blots

Total protein extraction from cells or ApoVs was performed with RIPA buffer (#P0013B, Beyotime) containing a protease inhibitor cocktail according to the manufacturer’s protocol, and the extracted protein was quantified with a BCA kit (#P0012, Beyotime). Nuclear and cytoplasmic proteins were extracted with a Nuclear and Cytoplasmic Protein Extraction Kit (#P0027, Beyotime) according to the manufacturer’s instructions. After the samples were subjected to SDS‒PAGE, they were transferred onto polyvinylidene fluoride (PVDF, Millipore) membranes. After being blocked with 5% skim milk for 1 h, the membranes were incubated with primary antibody at 4 °C overnight. After the membranes were washed 3 times with Tris-buffered saline with Tween (TBST), the membranes were incubated with secondary antibody (1:1000, #A0208, Beyotime) for 1 h at room temperature. Finally, a chemiluminescence system (Bio-Rad) was used to determine the protein expression level. The primary antibodies used in this study were as follows: caspase 3 (1:5000, #ab32351, Abcam), cleaved caspase 3 (1:500, #ab32042, Abcam), NAMPT (1:2000, #11776-1-AP, Proteintech), SIRT1 (1:1000, #13161-1-AP, Proteintech), acetyl-FOXO1 (Lys294) (1:500, #PA5-104560, Invitrogen), FOXO1 (1:1000, #AF1600, Beyotime), β-actin (1:5000, #81115-1-RR, Proteintech), and Lamin B1 (1:5000, #12987-1-AP, Proteintech).

### Statistical analysis

The experiments involved in this study were repeated at least 3 times. Statistical and graphical analyses were performed via SPSS (version 25.0). Significant differences between different groups were identified via Student’s t test and one-way ANOVA. All the data are presented as the means ± SEMs, and *P < 0.05 was considered to indicate statistical significance.

## Results

### Transplanted BMSCs undergo apoptosis, which ameliorates hind limb ischemia

We characterized the human BMSCs used in this study. As shown in Figure S1, the BMSCs grew in a vortex under a bright field (Figure S1A). FCM revealed that BMSCs positively expressed the MSC surface markers CD105, CD44, CD29, and CD90, while the hematopoietic stem cell surface markers CD45 and CD34 were not detected (Figure S1B).

**Fig. 1:**
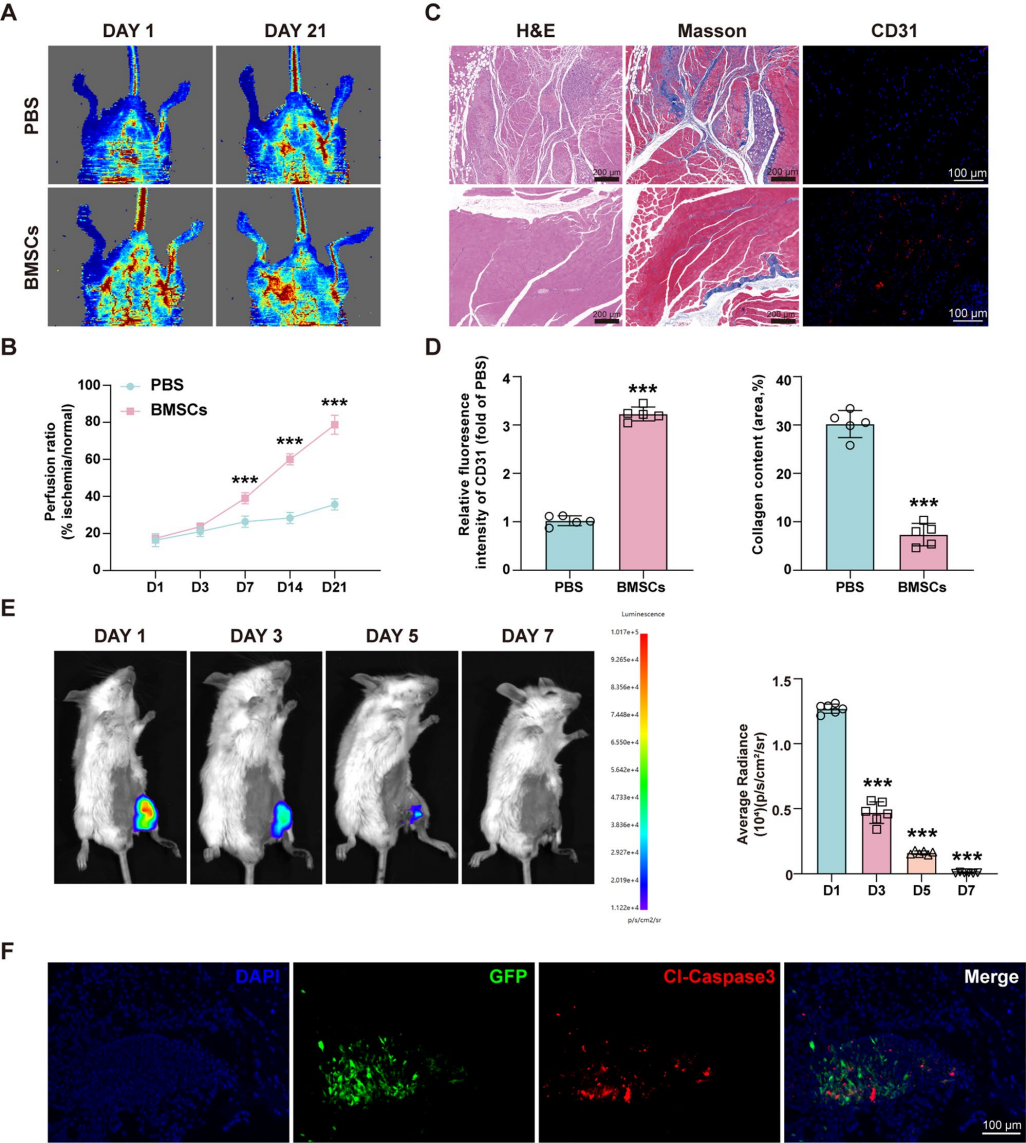
BMSCs undergo apoptosis in vivo after transplantation. **(A)** Representative laser Doppler perfusion images of the recovery of blood flow in ischemic hind limbs on day 0 and day 21 after intramuscular injection of PBS or BMSCs. **(B)** Blood perfusion ratios of the different groups at 21 days. (*n* = 6; ***P < 0.001 vs. PBS). **(C)** Representative images of gastrocnemius muscle sections subjected to H&E, Masson, and CD31 immunofluorescence staining in the PBS and BMSCs groups. **(D)** Quantitative analysis of CD31 fluorescence intensity (red) and Masson’s trichrome staining of collagen content (blue). (*n* = 5; ***P < 0.001 vs. PBS). **(E)** The survival of BMSCs^Fluc/eGFP^ was assessed via BLI. D-Luciferin potassium salt was injected intraperitoneally. (*n* = 6; ***P < 0.001 vs. Day 1). **(F)** Representative image of ischemic gastrocnemius muscle injected with BMSCs^Fluc/eGFP^ on Day 5, with a large accumulation of cleaved caspase 3 (red) near the BMSCs (green). Scale bars, 100 μm. All the data are representative of three independent experiments and are shown as means ± SEM

Subsequently, we performed intramuscular injections of either prepared PBS (PBS group) or BMSCs (BMSCs group) into the gastrocnemius muscle of mice with hind limb ischemia. Laser Doppler analysis revealed that the blood flow signals in the BMSCs group were stronger than the PBS group on Days 7, 14 and 21 (Fig. 1A and B). Additionally, histological analysis showed reduced infiltration of multinucleated cells and less vacuolar-like degeneration in the BMSCs group (Fig. 1C). Masson’s trichrome staining and CD31 immunofluorescence staining further demonstrated that BMSCs treatment significantly reduced collagen fiber content and promote the formation of microvessels (Fig. 1C and D). These findings demonstrate that BMSC transplantation can effectively improve blood flow perfusion and tissue damage in hind limb ischemia models.

To assess the survival of BMSCs in vivo after transplantation, we transfected BMSCs with lentiviruses encoding firefly luciferase and eGFP (Figure S1D). The survival of BMSCs^Fluc/eGFP^ was monitored via BLI on Days 1, 3, 5, and 7. The results revealed that the BMSC florescence signal began to weaken on Day 3 after injection and almost disappeared on Day 7 (Fig. 1E). Immunofluorescence staining of the gastrocnemius muscle on Day 5 revealed a significant presence of cleaved caspase-3 in the transplanted area, indicating the apoptosis of the BMSCs (Fig. 1F). Our data demonstrate that while transplanted BMSCs effectively ameliorate hind limb ischemia in mice, they undergo apoptosis shortly after transplantation.

### Isolated ApoVs promote tissue repair and angiogenesis in vivo

**Fig. 2:**
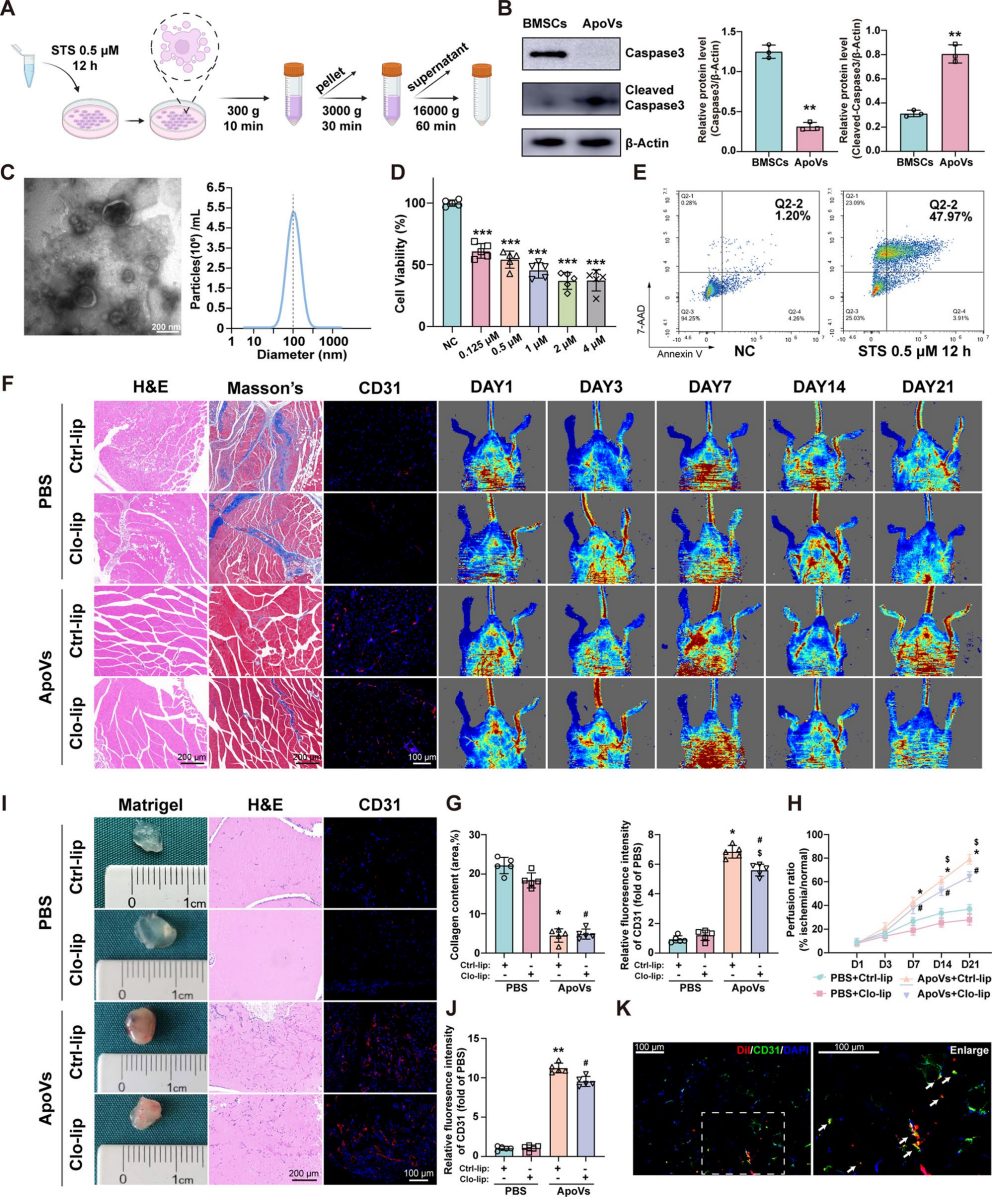
Isolated ApoVs promote tissue repair and angiogenesis in vivo. **(A)** Schematic diagram of the isolation of ApoVs. **(B)** Western blot analysis of caspase 3 and cleaved caspase-3 protein levels in ApoVs. (*n* = 3; **P < 0.01 vs. BMSCs) **(C)** TEM and NTA detection of the morphology and diameter of ApoVs. Scale bars, 100 nm. **(D)**&**(E)** STS-induced apoptosis of BMSCs was measured via CCK-8 and FCM assays. (*n* = 5; ***P < 0.001 vs. NC). **(F)** H&E, Masson, and CD31 fluorescence staining of the gastrocnemius muscle and laser Doppler results of hind limb ischemic mice treated with ApoVs (with clodronate liposomes or control liposomes). **(G)** Quantitative analysis of the collagen content and CD31 fluorescence intensity. (*n* = 5). **(H)** Blood perfusion ratios of the different groups at 21 days. (*n* = 6). **(I)** Representative photographs, H&E and CD31 fluorescence results of Matrigel plugs injected with ApoVs (with chlorophosphate liposomes or control liposomes) on Day 14. **(J)** Quantitative analysis of CD31 fluorescence in **(I)** (*n* = 5). **(K)** Representative image of colocalization of CD31 (green) and Dil-labeled ApoVs (red) in gastrocnemius muscle tissue. All data are shown as means ± SEM. (*P < 0.05 vs. PBS + Ctrl-lip; **P < 0.01 vs. PBS + Ctrl-lip; ^#^*P* < 0.05 vs. PBS + Clo-lip; ^$^*P* < 0.05 vs. ApoVs + Ctrl-lip)

To investigate whether the products generated from apoptotic BMSCs possess therapeutic effects, we induced BMSCs apoptosis by STS and assessed the degree of apoptosis via CCK-8 and FCM assays (Fig. 2D and E). Subsequently, ApoVs were isolated from the apoptotic BMSCs using differential centrifugation (Fig. 2A). Western blotting revealed that ApoVs exhibited high levels of cleaved caspase-3, while the levels of caspase-3 were comparatively low (Fig. 2B) (Full-length blots are presented in Additional file 2). NTA revealed that the diameters of the isolated ApoVs were predominantly around 100 nm (Fig. 2C). Furthermore, ApoVs exhibit a cup shape under TEM (Fig. 2C).

In the inflammatory environment of the transplant site, ApoVs is most likely to be absorbed by macrophages [21], which have been shown to regulate the process of angiogenesis [22]. To further investigate the therapeutic effects of ApoVs on hind limb ischemia, we first depleted macrophages in mice using clodronate liposomes. Immunofluorescence on Day 3 revealed a significant decrease in F4/80 fluorescence intensity across multiple organs (Figure S1C).

Next, we injected ApoVs into the gastrocnemius muscle and monitored the recovery process. Blood flow in the affected limbs of mice treated with ApoVs showed significant improvement on Days 7, 14, and 21, although the therapeutic effects were slightly diminished upon macrophage clearance (Fig. 2F and H). Additionally, we evaluated angiogenesis, tissue damage, and fibrosis in the gastrocnemius muscle. H&E staining revealed that the muscle tissue in the ApoVs group had a healthier morphology with reduced inflammatory cell infiltration (Fig. 2F). Masson’s staining and immunofluorescence showed reduced collagen deposition and a significant increase in CD31^+^ cells in the ApoVs group. (Figure 2F and G). Furthermore, clodronate liposomes only partially affected the above effects (Fig. 2F and G). We subsequently performed Matrigel plugs to verify the angiogenesis of ApoVs in vivo. The Matrigel containing either PBS or ApoVs was subcutaneously injected into mice with control liposomes or clodronate liposomes, respectively. The Matrigel plugs were collected for angiogenic analysis, more red blood vessels and tissues were present in the ApoVs group (Fig. 2I). H&E and immunofluorescence staining showed the presence of vascular-like structures containing blood cells and a significant increase in CD31-positive capillaries in the ApoVs group. Notably, the clearance of macrophages had no apparent impact on these observations (Fig. 2I and J).

Based on the above results, ApoVs promote the recovery of blood flow in mouse hind limb ischemia irrespective of macrophage depletion, suggesting that macrophages play a limited role in the therapeutic effects of ApoVs. To further investigate the mechanism of ApoVs, we labeled ApoVs with Dil and performed CD31 staining on the gastrocnemius muscle on Day 7. As shown in Fig. 2K, a substantial colocalization was observed between transplanted ApoVs and CD31-positive cells (white arrows in Fig. 2K). These findings suggest that the angiogenesis of transplanted ApoVs in vivo may primarily arise from their interactions with endothelial cells.

### ApoVs promote the biological function of HUVECs

**Fig. 3:**
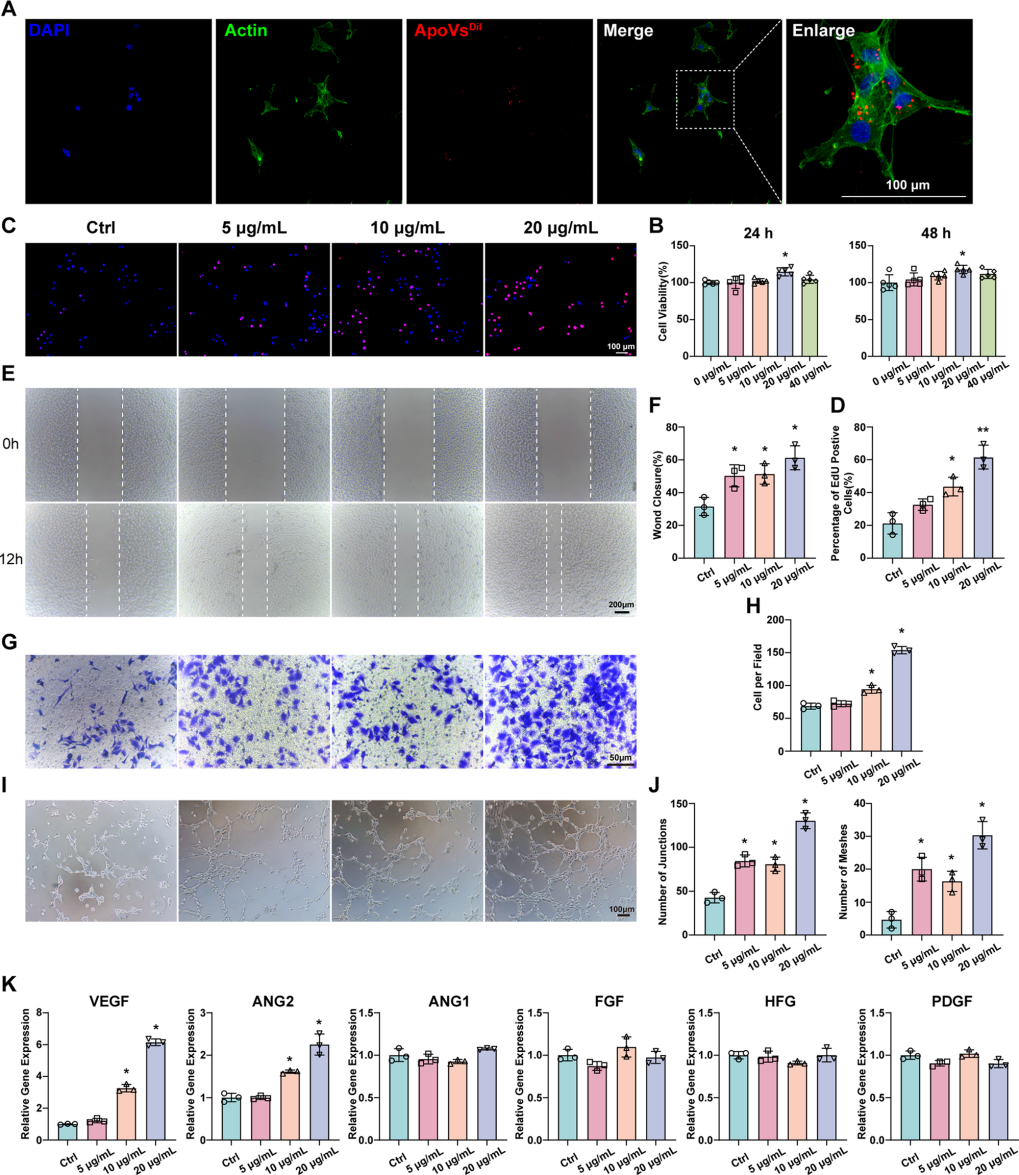
ApoVs promote the biological function of HUVECs. **(A)** Representative image of HUVECs internalization of Dil-labeled ApoVs. Scale bars, 100 μm. **(B)** CCK-8 analysis of HUVEC proliferation under the influence of different concentrations of ApoVs. (*n* = 5; *P < 0.05 vs. 0 µg/mL). **(C)** Representative images of EdU staining after treatment with different concentrations of ApoVs. Scale bars, 100 μm. **(D)** Quantitative analysis of the number of EdU-positive cells. **(E)** Representative images of the wound healing assay of HUVECs treated with different concentrations of ApoVs. Scale bars, 200 μm. **(F)** Quantitative analysis of the wound healing assay results. **(G)** Transwell assays were used to evaluate the migration of HUVECs treated with different concentrations of ApoVs. **(H)** Quantitative analysis of the Transwell assay results. **(I)** Representative images of the tube formation assay after treatment with different concentrations of ApoVs. **(J)** Quantitative analysis of junctions and meshes in the tube formation assay. **(K)** The expression of angiogenesis-related genes was examined via qRT‒PCR. Relative gene expression was calculated via the 2^−ΔΔCt^ method and normalized to GAPDH. All the data are representative of three independent experiments and are shown as means ± SEM. (*n* = 3; *P < 0.05 vs. Ctrl; **P < 0.01 vs. Ctrl)

In vivo data indicate that ApoVs colocalize with CD31, suggesting their potential interaction with endothelial cells. To further investigate this, we used HUVECs to study the effects of ApoVs on endothelial cell function. First, we confirmed the internalization of ApoVs by HUVECs. After coculturing HUVECs with Dil-labeled ApoVs (20 µg/mL) for 24 h, fluorescence imaging revealed the successful internalization of ApoVs into the cytoplasm of HUVECs (Fig. 3A).

The CCK-8 assay results demonstrated that ApoVs significantly enhanced the proliferation of HUVECs at both 24 and 48 h (Fig. 3B). EdU staining revealed that ApoVs increased the percentage of EdU-positive HUVECs (Fig. 3C and D). Furthermore, wound healing and Transwell assays revealed that ApoVs promote the migration of HUVECs in a concentration-dependent manner (Fig. 3E and H). Tube formation experiments revealed that ApoVs, especially at 20 µg/mL, significantly promoted the ability of HUVECs to form capillary-like tube structures (Fig. 3I and J). In addition, we analyzed the changes in the expression of angiogenesis-related genes via qPCR. The results revealed that VEGF and ANG2 were significantly upregulated after ApoVs treatment (Fig. 3K). In summary, these findings demonstrate that ApoVs can be internalized by HUVECs, subsequently enhancing their proliferation, migration, and angiogenic capabilities.

### ApoVs uptake by HUVECs depends on dynamin-, clathrin-, and caveolin-mediated endocytosis

**Fig. 4:**
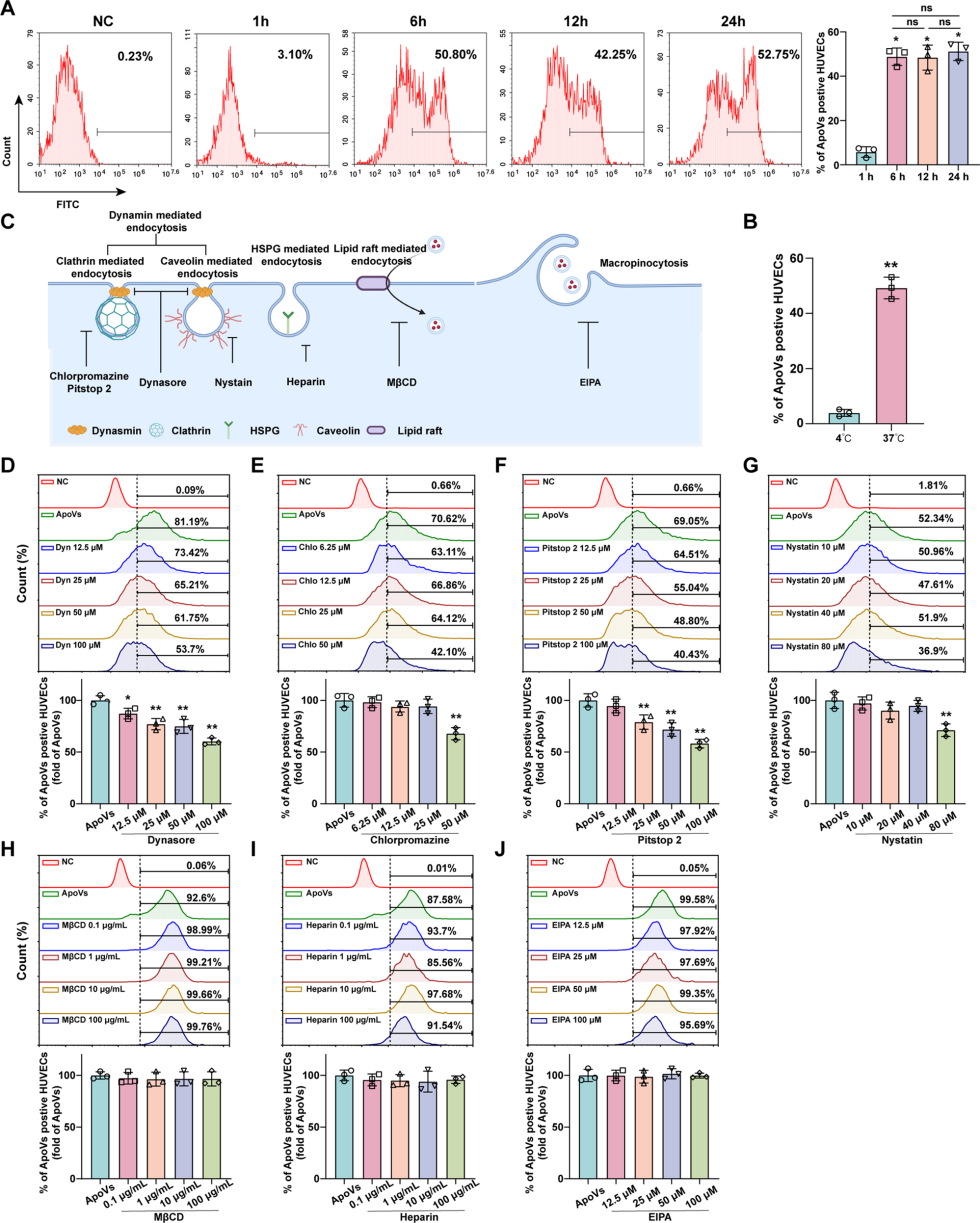
ApoVs uptake by HUVECs depends on dynamin-, clathrin-, and caveolin-mediated endocytosis. The effects of **(A)** incubation time and **(B)** temperature on the internalization of DiO-ApoVs in HUVECs treated with 20 µg/mL ApoVs. **(C)** Schematic diagram of the endocytosis pathway in HUVECs. **(D)** Percentages of HUVECS positive for DiO-ApoVs in the presence of increasing concentrations of dynasore (12.5, 25, 50, and 100 µM), **(E)** chlorpromazine (6.25, 12.5, 25, and 50 µM), **(F)** Pitstop 2 (12.5, 25, 50, and 100 µM), **(G)** NYST (10, 20, and 40 80 µM), **(H)** MβCD (0.1, 1, 10, and 100 µg/mL), **(I)** heparin (0.1, 1, 10, and 100 µg/mL) and **(J)** EIPA (12.5, 25, 50, and 100 µM). All the data are representative of three independent experiments and are presented as the means ± SEMs. (*n* = 3; *P < 0.05 vs. ApoVs; **P < 0.01 vs. ApoVs)

Previous studies have confirmed that ApoVs promote the biological functions of HUVECs. To further investigate the mechanism of the internalization, we labeled ApoVs with the cell membrane dye DiO and used FCM to analyze their uptake by HUVECs. We discovered that 20 µg/mL ApoVs reached peak uptake efficiency at 6 h, after which extending the incubation time did not further increase the uptake rate. (Fig. 4A). Moreover, ApoVs uptake was completely inhibited at 4 °C, indicating an active, energy-dependent process (Fig. 4B). To further elucidate the relationship between specific endocytotic processes and ApoVs, we pretreated HUVECs with different inhibitors. First, we assessed the effects of these inhibitors on HUVEC viability to ensure that the selected concentrations did not compromise cell health (Figure S2A). Dynamin is a major participant in cellular internalization and participates in endocytosis mediated by clathrin and caveolin [23, 24] (Fig. 4C). After we blocked dynamin with the specific inhibitor dynasore, the uptake of ApoVs by HUVECs significantly decreased (Fig. 4D). To further differentiate endocytic routes, we further employed chlorpromazine and Pitstop 2 to block clathrin and NYST to block caveolin. The results revealed that both the clathrin- and caveolin-mediated endocytosis contribute to ApoVs internalization (Fig. 4E and G). In addition, we observed the involvement of other pathways, including lipid rafts, heparan sulfate proteoglycans (HSPG), and macrophagosomes. The inhibitors targeting these pathways were unable to block the uptake of ApoVs (Fig. 4H and J). In summary, our results indicate that dynamin-, clathrin-, and caveolin-mediated endocytosis are involved in the uptake of ApoVs by HUVECs.

### Proteomic analysis of HUVECs and HUVECs-ApoVs

**Fig. 5:**
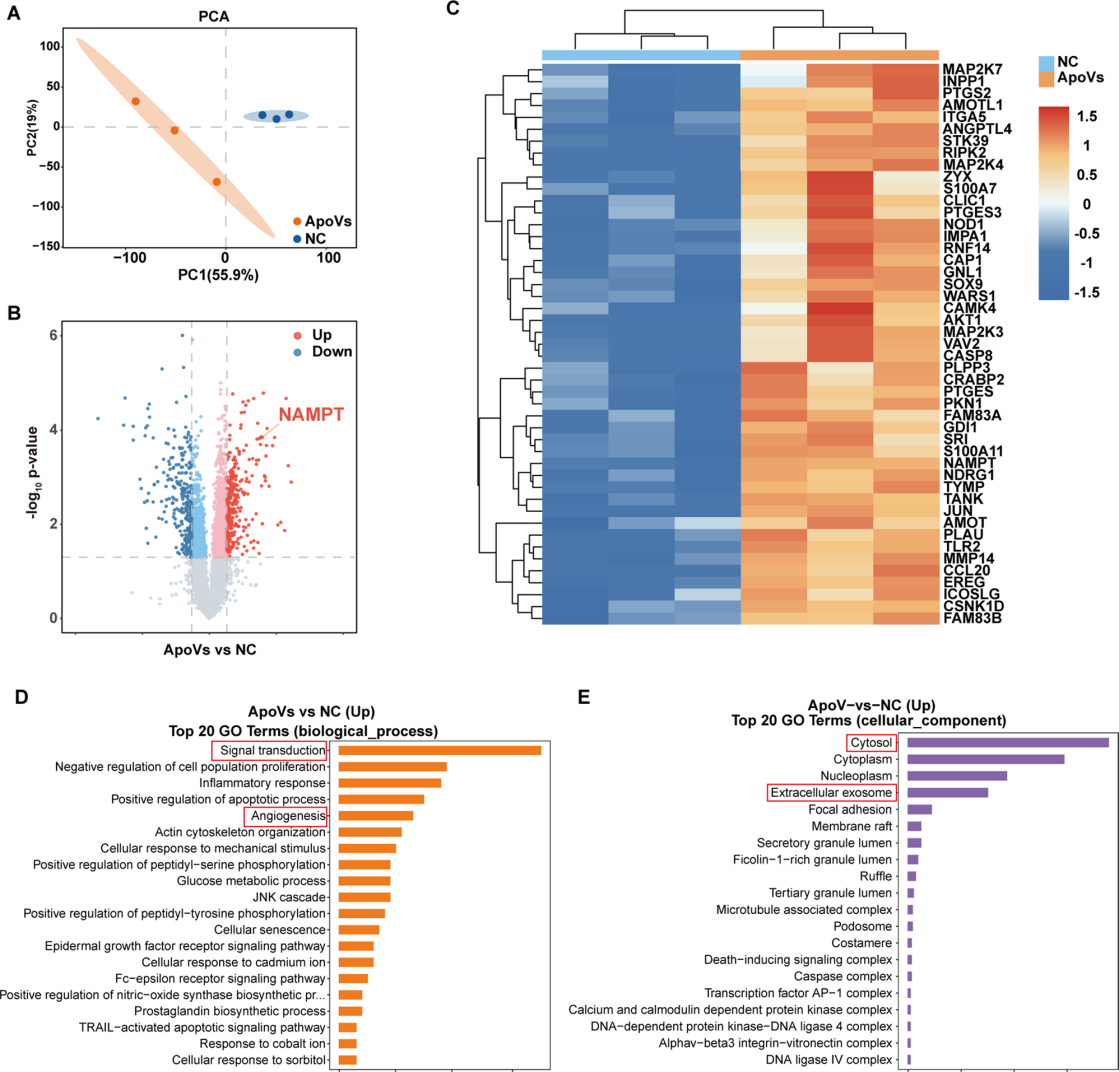
Proteomic analysis of HUVECs and HUVECs-ApoVs. **(A)** PCA of proteomic data. PCA1 and PCA2 represent the largest sources of variation. **(B)** Volcano plots of differentially expressed proteins in HUVECs before and after ApoVs intervention. **(C)** Heatmap of differentially expressed proteins related to signal transduction and angiogenesis. **(D)** GO analysis of the significantly differentially expressed proteins in ApoVs and the top 20 genes associated with **(D)** biological processes and **(E)** cellular components

To investigate the molecular mechanism underlying the effects of ApoVs on HUVECs, we performed TMT-based proteomic analysis to compare protein expression profiles between control and ApoVs-treated HUVECs. Principal component analysis (PCA) revealed a total variation of 55.9% between the two sample groups (Fig. 5A). In total, 7251 proteins were identified. Differentially expressed proteins were selected on an absolute fold change of ≥ 1.2 and a P value < 0.05. This analysis identified 607 proteins with significant expression expressed between the NC and ApoVs groups, of which 315 were upregulated and 292 were downregulated (Fig. 5B). To further explore the biological processes associated with these differentially expressed proteins, we conducted bioinformatic enrichment analysis. GO database analysis indicated that the upregulated proteins were strongly associated with biological processes such as “signal transduction”, “angiogenesis”, “cytoplasm”, and “extracellular vesicles” (Fig. 5D). A clustering heatmap provided a clearer visualization of upregulated proteins involved in signal transduction and angiogenesis (Fig. 5C). Additionally, KEGG enrichment analysis demonstrated that ApoVs treatment enhanced the expression of proteins involved in pathways related to metabolism, inflammation, immunity, Toll-like receptors, and Nod-like receptors (Figure S3C).

In the above results, the expression of NAMPT, a rate-limiting enzyme in the biosynthesis of nicotinamide adenine dinucleotide (NAD) metabolism, was significantly elevated in ApoVs-treated HUVECs (Fig. 5B). NAMPT was closely linked to processes such as signal transduction and angiogenesis, suggesting its pivotal role in mediating ApoVs-induced effects.

### NAMPT derived from ApoVs promote biological function of HUVECs via the NAMPT/SIRT1/FOXO1 axis

**Fig. 6:**
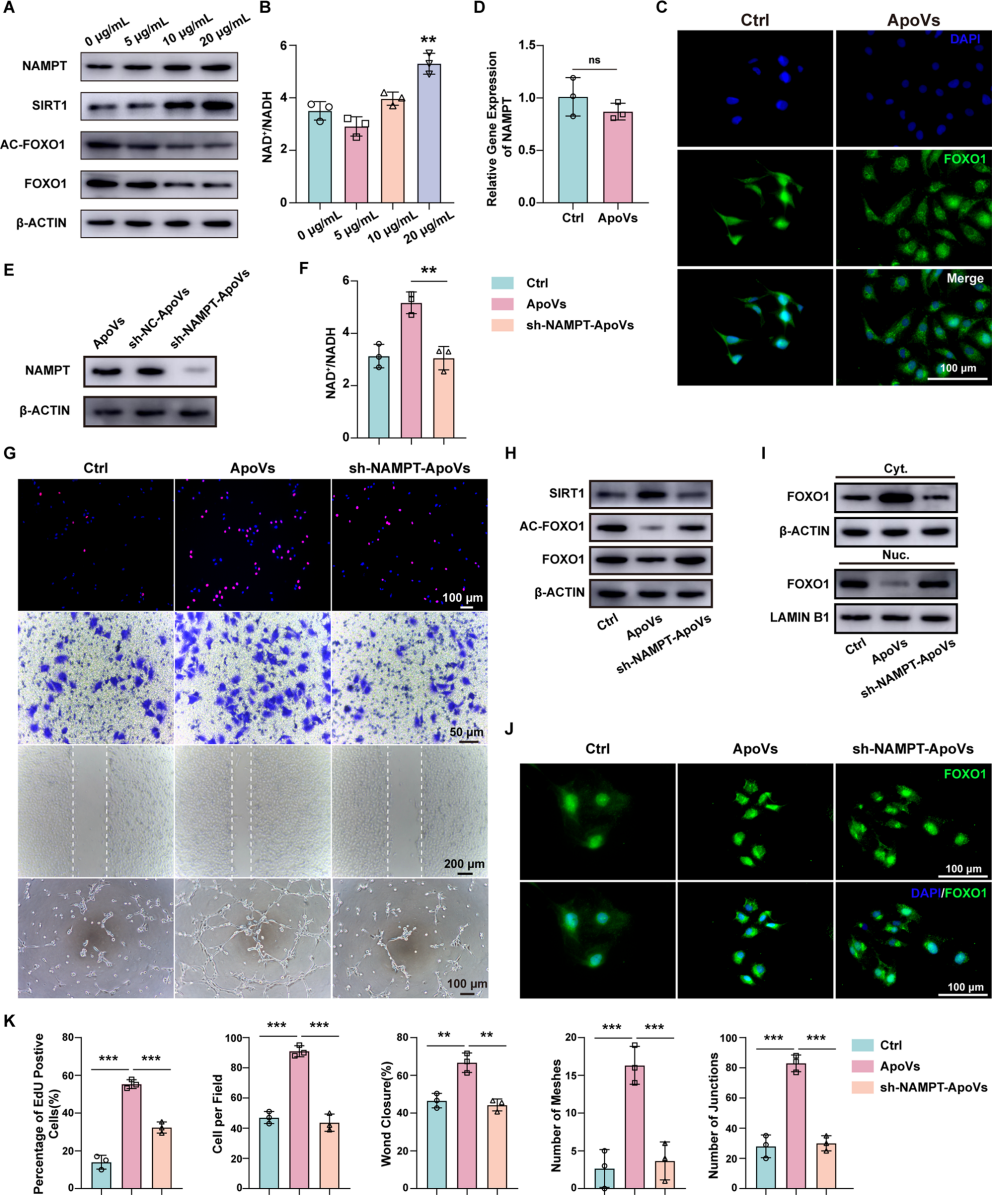
NAMPT derived from ApoVs promote biological function of HUVECs via the NAMPT/SIRT1/FOXO1 axis. **(A)** Western blotting revealed that NAMPT and SIRT1 increased while AC-FOXO1 and FOXO1 decreased in HUVECs after incubation with ApoVs (0, 5, 10, or 20 µg/mL). **(B)** NAD^+^/NADH ratio in HUVECs after incubation with ApoVs (0, 5, 10, or 20 µg/mL) (*n* = 3; **P < 0.01 vs. 0 µg/mL). **(C)** Representative image of FOXO1 immunofluorescence staining in HUVECs. Scale bars, 100 μm. **(D)** qRT‒PCR results of NAMPT after treating HUVECs with ApoVs. **(E)** Western blotting for detecting NAMPT in ApoVs, sh-NC-ApoVs and sh-NAMPT-ApoVs. **(F)** NAD+/NADH ratios in the Ctrl, ApoVs and sh-NAMPT-ApoVs groups. **(G)** Representative images of EdU, Transwell, wound healing and tube formation assays in the Ctrl, ApoVs and sh-NAMPT-ApoVs groups. **(H)** Western blotting of NAMPT, SIRT1, AC-FOXO1 and FOXO1 in the Ctrl, ApoVs and sh-NAMPT-ApoVs groups. **(I)** Western blot analysis of FOXO1 in the cytoplasm (Cyt.) and nucleus (Nuc.) in Ctrl, ApoVs and sh-NAMPT-ApoVs. **(J)** Representative image of FOXO1 immunofluorescence staining in the Ctrl, ApoVs and sh-NAMPT-ApoVs groups. Scale bars, 100 μm. **(K)** Quantitative analysis of EdU, Transwell, wound healing, and tube formation assays in **(G)**. All the data are representative of three independent experiments and are shown as means ± SEMs (*n* = 3; **P < 0.01; ***P < 0.001; ns *P* > 0.05)

NAMPT can regulate the activity of its key downstream molecule SIRT1 by modulating the NAD^+^/NADH ratio. To confirm the upregulation of NAMPT in HUVECs, we treated the cells with varying concentrations of ApoVs (0 µg/mL, 5 µg/mL, 10 µg/mL, or 20 µg/mL) for 24 h. Western blot analysis showed that NAMPT expression levels increased in a concentration-dependent manner with the increasing amount of ApoVs (Fig. 6A) (Full-length blots are presented in Additional file 2). Similar to NAMPT, the intracellular NAD^+^/NADH ratio also increased correspondingly (Fig. 6B). Additionally, the expression of SIRT1 was elevated, while the levels of acetyl-FOXO1 and FOXO1 were reduced (Fig. 6A). FOXO1, a negative regulator of angiogenesis, has its transcriptional activity influenced by acetylation. To further investigate, we examined the subcellular localization of FOXO1 using immunofluorescence staining. In the control group, FOXO1 was predominantly localized in the nucleus; however, in the ApoVs group, FOXO1 was distributed in the cytoplasm (Fig. 6C). These results indicate that treatment with ApoVs prompted FOXO1 in HUVECs to translocate into the cytoplasm, thereby diminishing its transcriptional activity.

Our previous data demonstrated that treating HUVECs with ApoVs led to an upregulation of NAMPT. To investigate the underlying mechanism of this upregulation, we first used qPCR to analyze the mRNA expression levels in HUVECs. Interestingly, the addition of ApoVs did not result in an increase in NAMPT mRNA expression (Fig. 6D), indicating that the upregulation of NAMPT is not driven by transcriptional activation. Based on this, we hypothesize that NAMPT is transferred to HUVECs via ApoVs, thereby initiating a series of downstream effects. This hypothesis was further supported by western blot analysis, which confirmed the presence of NAMPT in ApoVs (Fig. 6E).

To further explore the role of NAMPT, we downregulated its expression by transfecting BMSCs with short hairpin RNA (shRNA) targeting NAMPT, followed by isolation of ApoVs. After confirming the effective downregulation of NAMPT (Fig. 6E), we treated HUVECs with two types of ApoVs (ApoVs and sh-NAMPT-ApoVs) and compared them to untreated HUVECs as controls. Notably, the downregulation of NAMPT in the ApoVs resulted in a decrease in the NAD^+^/NADH ratio, a reduction in SIRT1 levels, and an increase in both acetylated FOXO1 (AC-FOXO1) and FOXO1 protein levels (Fig. 6F and H). Functionally, this decrease in NAMPT also led to reduced proliferation, migration, and angiogenesis of HUVECs (Fig. 6G and K). Furthermore, we assessed the subcellular distribution of FOXO1. In the ApoVs group, FOXO1 levels were markedly decreased in the nucleus, accompanied by a corresponding increase in the cytoplasm. However, in the sh-NAMPT-ApoVs group, this phenomenon was reversed. (Fig. 6I) (Full-length blots are presented in Additional file 2). Immunofluorescence staining further validated the redistribution of FOXO1, confirming these findings (Fig. 6J).

In conclusion, these findings demonstrate that ApoVs regulate the subcellular localization of FOXO1 and promote biological functions of HUVECs by delivering NAMPT.

### ApoVs promote the biological function of HUVECs via SIRT1

**Fig. 7:**
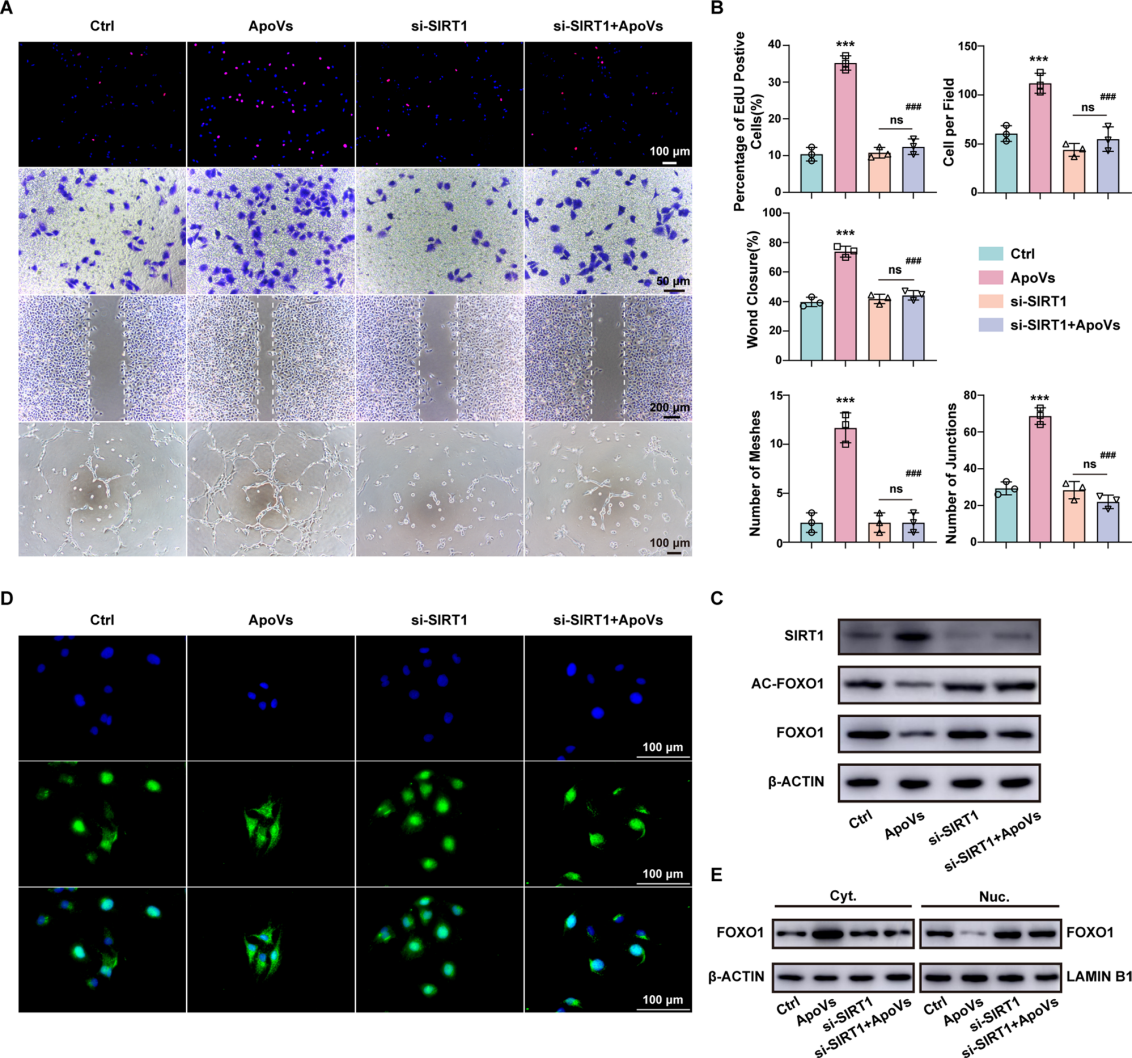
ApoVs promote the biological function of HUVECs via SIRT1. **(A)** Representative images of EdU, Transwell, wound healing and tube formation assays in the Ctrl, ApoVs, si-SIRT1 and si-SIRT1 + ApoVs groups. **(B)** Quantitative analysis of the results of the EdU, Transwell, wound healing, and tube formation assays in (A). **(C)** Western blotting of SIRT1, AC-FOXO1 and FOXO1 in the Ctrl, ApoVs, si-SIRT1 and si-SIRT1 + ApoVs groups. **(D)** Representative image of FOXO1 immunofluorescence staining in the Ctrl, ApoVs, si-SIRT1 and si-SIRT1 + ApoVs groups. **(E)** Western blotting of FOXO1 in the cytoplasm (Cyt.) and nucleus (Nuc.) in the Ctrl, ApoVs, si-SIRT1 and si-SIRT1 + ApoVs groups. All the data are representative of three independent experiments and are shown as the means ± SEMs. (*n* = 3; ***P < 0.001 vs. Ctrl; ^###^*P* < 0.001 vs. ApoVs; ns *P* > 0.05)

Several studies have shown that NAMPT can affect the expression of SIRT1 through NAD^+^/NADH [25, 26]. SIRT1, an NAD^+^-dependent deacetylase, is involved in transcriptional regulation, cell survival, and energy metabolism [27, 28]. To confirm the effect of SIRT1 on angiogenesis, we employed specific siRNAs to knock down SIRT1 (Figure S2B). The results showed that si-SIRT1 significantly inhibited the proliferation, migration, and angiogenesis of HUVECs stimulated by ApoVs (Fig. 7A and B). Western blot analysis further revealed that knocking down SIRT1 reversed the reduction in acetyl-FOXO1 and FOXO1 levels induced by ApoVs. In the si-SIRT1 + ApoVs group, ApoVs failed to downregulate acetyl-FOXO1 and FOXO1, underscoring the pivotal role of SIRT1 in mediating the biological effects of ApoVs (Fig. 7C) (Full-length blots are presented in Additional file 2). Finally, we validated the alterations in FOXO1 subcellular localization through immunofluorescence and nuclear protein analysis. In the APOV group, the expression level of FOXO1 decreases in the nucleus and increases in the cytoplasm, while knocking down SIRT1 eliminates this change. In the ApoV group, FOXO1 levels decreased in the nucleus while increasing in the cytoplasm. However, in si-SIRT1 + ApoVs group, the knockdown of SIRT1 abolished the effect of ApoV on FOXO1 localization (Fig. 7E). (Full-length blots are presented in Additional file 2). Consistently, immunofluorescence analysis confirmed this phenomenon (Fig. 7D).

These findings suggest that ApoVs promote the proliferation, migration, and angiogenesis of HUVECs by regulating FOXO1 localization via SIRT1, highlighting the central role of SIRT1 in mediating ApoVs’ biological effects.

## Discussion

The use of MSCs in the field of angiogenesis has a long-established history. MSCs derived from various tissues, including adipose tissue, bone marrow, dental pulp, umbilical cord, and skin, have been demonstrated to promote angiogenesis through mechanisms such as paracrine, transdifferentiation, and immunomodulatory effects [29–32]. In our animal experiments, transplanted MSCs were metabolized within a short time, yet their therapeutic effects were sustained for a notable period. Unfortunately, previous studies have been unable to provide an explanation for this phenomenon.

In the past, ApoVs were thought to be merely waste or harmful byproducts of apoptosis. However, it is now recognized that they play a crucial role in intercellular communication. Studies have shown that cells release EVs during apoptosis, which facilitate tissue regeneration by transferring biological molecules such as proteins, RNA, and DNA from parental cells [33–36]. Unlike common exosomes, ApoVs are easier to isolate and can be obtained in larger quantities [37], making them a promising candidate for therapeutic applications. In this study, we focused on the ApoVs of BMSCs. By inducing BMSCs apoptosis in vitro and isolating ApoVs for injection therapy, we observed that independently injected ApoVs demonstrated a significant therapeutic effect on ischemic hind limbs. This finding further validates the therapeutic potential of ApoVs.

To explore the in vivo mechanism of action of ApoVs, we depleted macrophages and found that they had a minimal effect on the therapeutic process of ApoVs. Subsequently, through co-localization of CD31 and ApoVs, we discovered that HUVECs internalize ApoVs, which enhances their biological function. Current research indicates that EVs reach target cells and initiate signal transduction primarily through three mechanisms: (1) binding to receptors on the surface of target cells, (2) fusion with the plasma membrane, and (3) internalization by target cells. Among these, receptor-mediated internalization is identified as the primary pathway through which EVs enter cells [38, 39]. In this study, we examined six distinct endocytosis mechanisms using various inhibitors, including dynamin-, clathrin-, caveolin-, HSPG-, lipid rafts-mediated endocytosis, and macrophagosomes. Our findings demonstrate that ApoVs bind to cell surface receptors and are internalized through dynamin-, clathrin-, and caveolin-mediated endocytosis.

To investigate the pathway through which ApoVs enhance the biological function of HUVECs, we conducted proteomic sequencing and observed a significant increase in NAMPT protein levels. NAMPT, also referred to as visfatin, serves as the rate-limiting enzyme in the biosynthesis of NAD [40]. By regulating the NAD pool, NAMPT can influence the activity of NAD-dependent enzymes, including sirtuins [25]. Our study demonstrated that ApoVs elevate intracellular NAD + levels, increase SIRT1 expression, and reduce the levels of AC-FOXO1 and FOXO1 by delivering NAMPT. The vascular endothelium is a critical physiological target of both FOXO and sirtuin family members, which play essential roles in regulating various aspects of vascular development and function. Previous reports have shown that the SIRT1/FOXO1 axis contributes to hippocampal angiogenesis in depression treatment [41] and facilitates wound healing in diabetic models [42]. Additionally, FOXO1 has been considered as a negative regulator of angiogenesis [43–45], with its activity modulated by post-translational modifications. Our study revealed that ApoVs enhance SIRT1 expression, which in turn promotes the deacetylation of FOXO1. This deacetylation causes FOXO1 to translocate from the nucleus to the cytoplasm, thereby reducing its inhibitory effect on angiogenesis. Currently, the role of acetylation in regulating FOXO1 activity remains incompletely understood. Some studies have reported that SIRT1-mediated deacetylation increases FOXO1’s transcriptional activity [46–48], while others suggest that deacetylation decreases its activity [42, 49–51]. In our research, we observed that FOXO1’s transcriptional activity diminishes following deacetylation, providing novel evidence for the regulatory role of acetylation in FOXO1 function.

Although our study demonstrated that ApoVs promoted the biological function of HUVECs and alleviated hind limb ischemia in mice, several challenges remain regarding their clinical application. First, the composition of ApoVs is complex and heterogeneous, requiring the development of standardized methods for separation and extraction. Previous studies have shown functional differences among various ApoV subtypes [52], highlighting the need for a standardized extraction process to ensure quality control. Second, angiogenesis is regulated by multiple interconnected signaling pathways. While our study identified certain mechanisms through which ApoVs promote angiogenesis, further research is needed to explore additional pathways that may also play a role. Finally, the ApoVs involved in this study are exogenous ApoVs, and the role and distribution of endogenous ApoVs still need to be further studied.

## Conclusion

Our research demonstrated that BMSCs have a prolonged therapeutic effect after being transplanted into ischemic hind limbs, primarily due to the release of NAMPT-containing ApoVs during apoptosis in vivo. These ApoVs are taken up by HUVECs and promote their proliferation, migration, and angiogenesis, possibly through the NAMPT/SIRT1/FOXO1 axis. This study provides different views on the treatment of CLTI by BMSCs and provides a promising direction for ApoVs-based therapy.

## Electronic supplementary material

Below is the link to the electronic supplementary material.

Supplementary Material 1

Supplementary Material 2

## Data Availability

The dataset generated during the present study is available upon reasonable request from the corresponding authors. The data reported in this paper have been deposited in the OMIX, China National Center for Bioinformation / Beijing Institute of Genomics, Chinese Academy of Sciences (https://ngdc.cncb.ac.cn/omix: accession no.OMIX008509).

## Abbreviations

ApoVs: Apoptotic vesicles
EVs: Extracellular vesicles
BMSCs: Bone marrow mesenchymal stem cells
HUVECs: Human umbilical vein endothelial cells
CLTI: Chronic limb-threatening ischemia
NAMPT: Nicotinamide phosphoribosyl transferase
SIRT1: Sirtuin 1
FOXO1: Forkhead box O1
MSCs: Mesenchymal stem cells
PAD: Peripheral arterial disease
H&E: Hematoxylin and eosin
OCT: Optimal cutting temperature
DAPI: 4’-6-diamidino-2-phenylindole
BLI: Bioluminescence imaging
ATCC: American type culture collection
FCM: Flow cytometry
TEM: Transmission electron microscopy
NTA: Nanoparticle tracking analysis
CCK-8: Cell counting kit-8
NYST: Nystatin
EIPA: Ethylisopropylamiloride
MβCD: Methyl-β-cyclodextrin
STS: Staurosporine
TMT: Tandem mass tag
