# Supplementary material for: Apoptotic vesicles derived from bone marrow mesenchymal stem cells increase angiogenesis in a hind limb ischemia model via the NAMPT/SIRT1/FOXO1 axis
Source: Stem Cell Res Ther. 2025 Mar 1;16:105. doi: 10.1186/s13287-025-04245-1 (PMC11872336; doi:10.1186/s13287-025-04245-1)
Supplement: Supplementary file 1 — Supplementary Material 1 [file 13287_2025_4245_MOESM1_ESM.docx]

**Supplementary Material**

**Apoptotic vesicles derived from bone marrow mesenchymal stem cells increase angiogenesis in a hind limb ischemia model via the NAMPT/SIRT1/FOXO1 axis**

Jinxing Chen^1#^, Zekun Shen^1#^, Bingyi Chen^1^, Shaung Liu^1^, Yifan Mei^1^, Kai Li^2^, Ziyang Peng^3^, Chaoshuai Feng^4^, Weiyi Wang^1^* and Shaoying Lu^1^*

^1^Department of Vascular Surgery, the First Affiliated Hospital of Xi’an JiaoTong University, Xi’an, Shaanxi Province, 710061, P.R. China

^2^Department of Otorhinolaryngology-Head and Neck Surgery, the First Affiliated Hospital of Xi’an Jiaotong University, Xi’an, Shaanxi Province, 710061, P.R. China

^3^School of Future Technology, National Local Joint Engineering Research Center for Precision Surgery & Regenerative Medicine, Xi'an Jiaotong University, Xi'an, Shaanxi Province, 710061, China

^4^Department of Spine Surgery, Hong Hui Hospital, Xi'an Jiaotong University, 555 You Yi Dong Road, Xi'an, Shaanxi Province,710054, P.R. China

# These authors contributed equally to this work

* Correspondence: robertlu@mail.xjtu.edu.cn


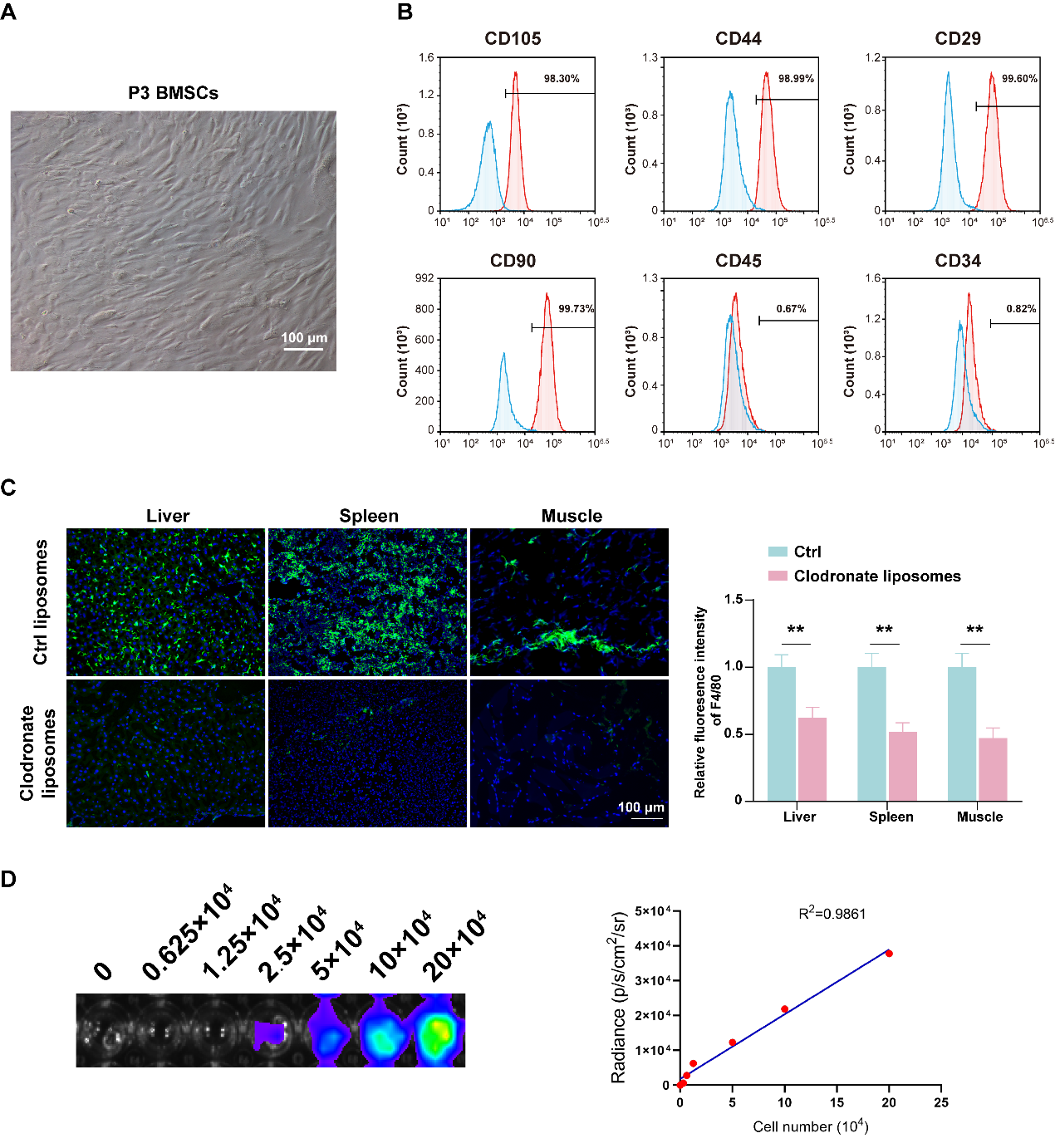


Figure S1. **Identification of BMSCs and mouse tissue F4/80 immunofluorescence staining.** (A) Morphology of BMSCs under optical microscope. (B) FCM was used to detect surface markers of BMSCs. (C) Representative images of F4/80 immunofluorescence in mouse liver, spleen, and muscle. (D) The transfection of the Fluc/eGFP gene was observed under BLI.


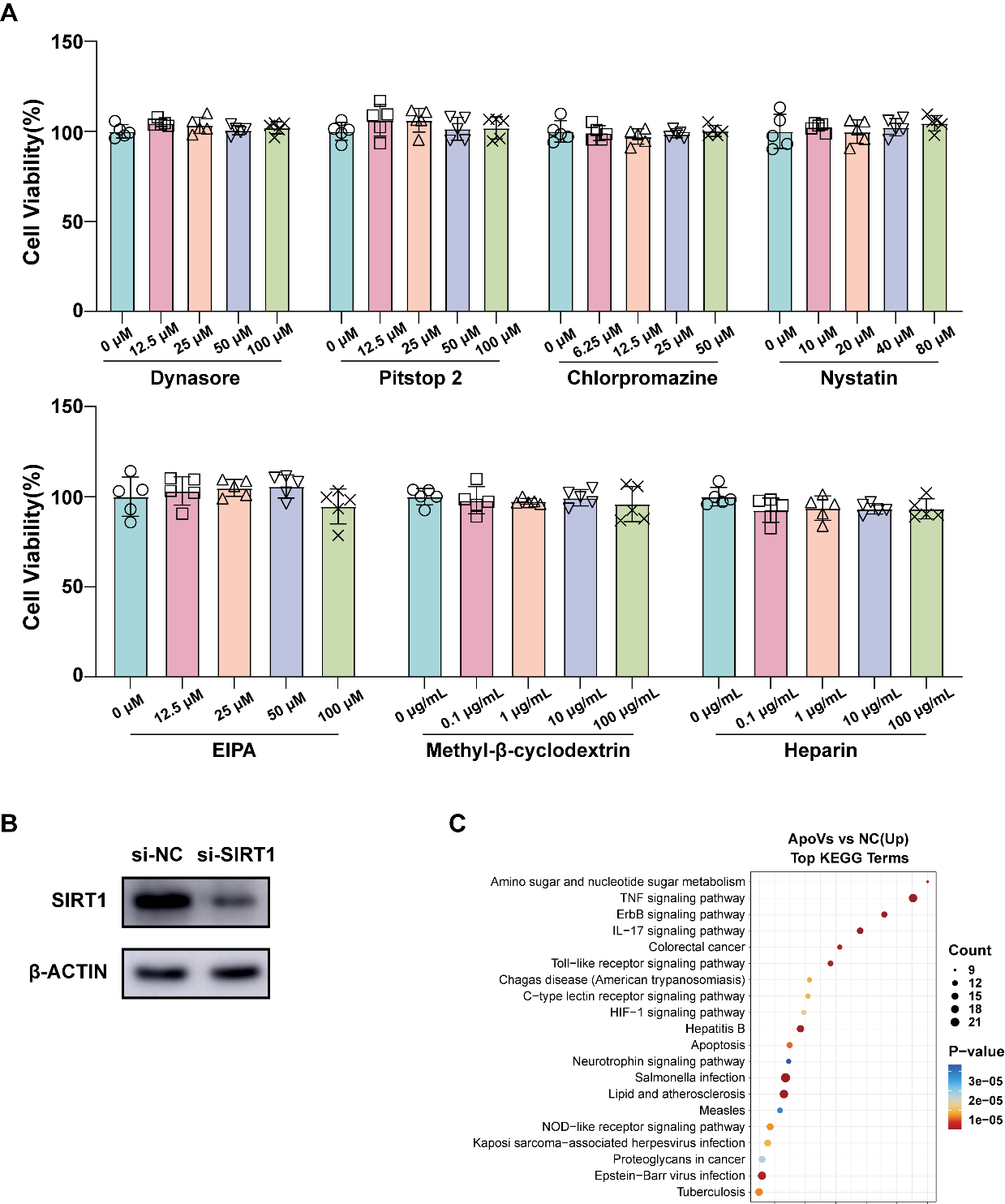


Figure S2 **Cell survival rate of HUVECs treated with inhibitors, validation of si-SIRT1 knockout efficiency and KEGG analysis of ApoVs treated HUVECs.** (A) Cells were pretreated with complete culture medium for 30 minutes containing dynasore (0-100 μM), chlorpromazine (0-50 μM), Pitstop 2 (0-100 μM), nystatin (0-80 μM), EIPA (0-100 μM), MβCD (0-100 μg/mL) and heparin (0-100 μg/mL). (B) Si-SIRT1 knockdown efficiency verification. (C) KEGG enrichment analysis of upregulate gene.

**Supplementary Table1 Primers in the qPCR assay**

| Gene | 5’-3’ |
| --- | --- |
| *VEGFA* F | AGGGCAGAATCATCACGAAGT |
| *VEGFA* R | AGGGTCTCGATTGGATGGCA |
| *HGF* F | GCTATCGGGGTAAAGACCTACA |
| *HGF* R | GGTCCTGGGTATTGGAGCA |
| *FGF-2* F | CAAAAACGGGGGCTTCTTCC |
| *FGF-2* R | GTCGCTCTTCTCCCGGAC |
| *PDFG* F | GCAAGACCAGGACGGTCATTT |
| *PDFG* R | GGCACTTGACACTGCTCGT |
| *ANG1* F | AGAACCTTCAAGGCTTGGTTAC |
| *ANG1* R | GGTGGTAGCTCTGTTTAATTGCT |
| *ANG2* F | CTCGAATACGATGACTCGGTG |
| *ANG2 R* | TCATTAGCCACTGAGTGTTGTTT |
| NAMPT F | CGGCAGAAGCCGAGTTCAA |
| NAMPT R | GCTTGTGTTGGGTGGATATTGTT |
| *GAPDH* F | GGAGCGAGATCCCTCCAAAAT |
| *GAPDH* R | GGCTGTTGTCATACTTCTCATGG |

**Supplementary Table2 Sequences of shRNA and siRNA**

| Gene | 5’-3’ |
| --- | --- |
| *ShRNA-NC* |  |
|  | GATCCGTTCTCCGAACGTGTCACGTAATTCAAGAGATTACGTGACACGTTCGGAGAATTTTTTC |
|  |  |
|  |  |
|  | AATTGAAAAAATTCTCCGAACGTGTCACGTAATCTCTTGAATTACGTGACACGTTCGGAGAACG |
|  |  |
| *ShRNA-Nampt* |  |
|  | GATCCGTAACTTAGATGGTCTGGAATCTCGAGATTCCAGACCATCTAAGTTACTTTTTTG |
|  |  |
|  |  |
|  | AATTCAAAAAAGTAACTTAGATGGTCTGGAATCTCGAGATTCCAGACCATCTAAGTTACG |
|  |  |
| *Si-NC* |  |
|  | UUCUCCGAACGUGUCACGUTT |
|  |  |
|  | ACGUGACACGUUCGGAGAATT |
| *Si-SIRT1* |  |
|  | GAAAUUAUCACUAAUGGUUUU |
|  |  |
|  | AACCAUUAGUGAUAAUUUCAU |
|  |  |
