# Supplementary material for: Apoptotic vesicles derived from bone marrow mesenchymal stem cells increase angiogenesis in a hind limb ischemia model via the NAMPT/SIRT1/FOXO1 axis
Source: Stem Cell Res Ther. 2025 Mar 1;16:105. doi: 10.1186/s13287-025-04245-1 (PMC11872336; doi:10.1186/s13287-025-04245-1)

The images of western blotting in Fig 2B.


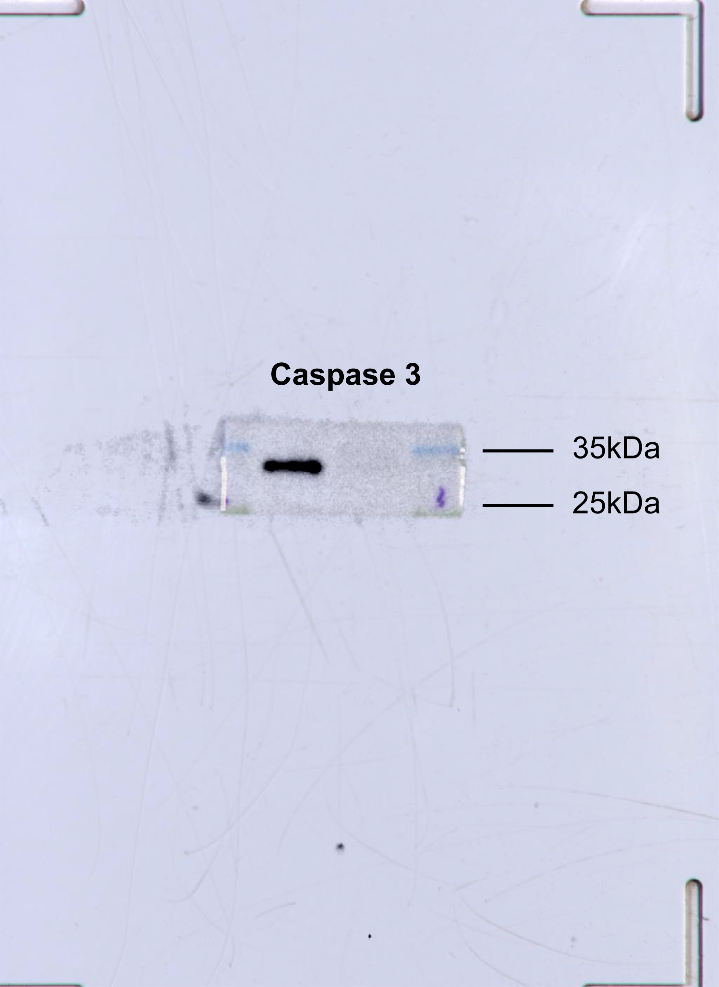


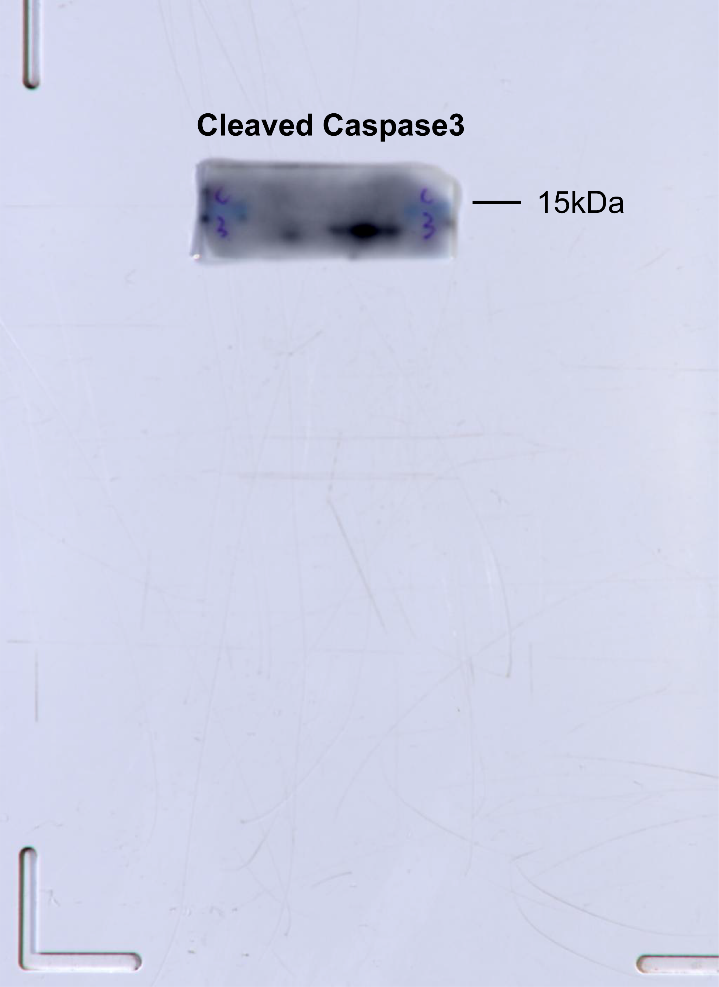

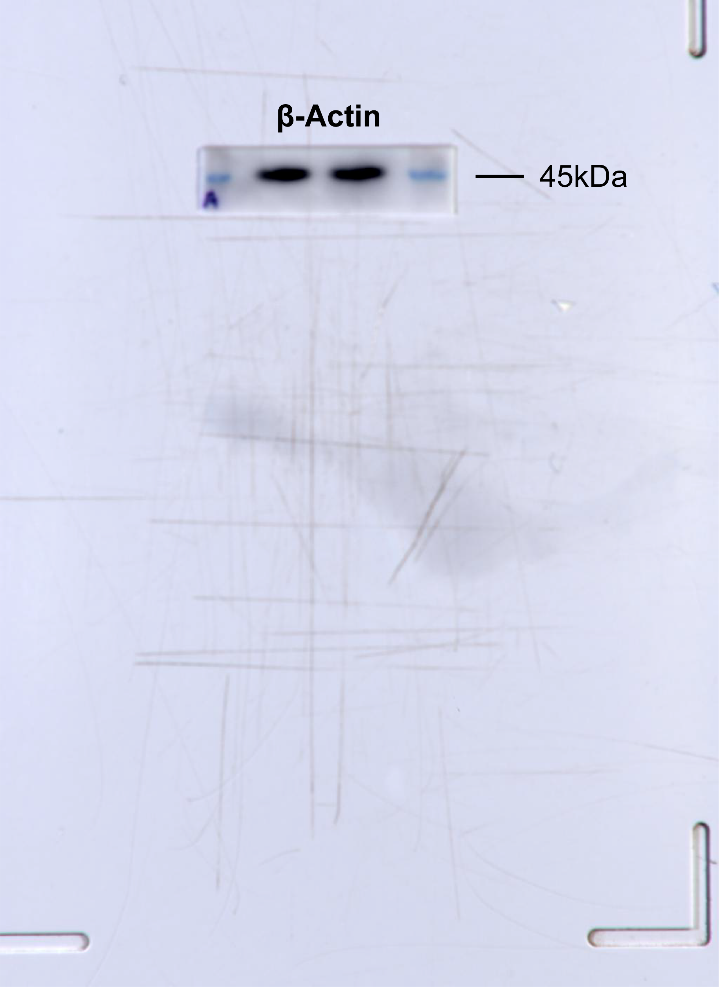


The images of western blotting in Fig 6A.


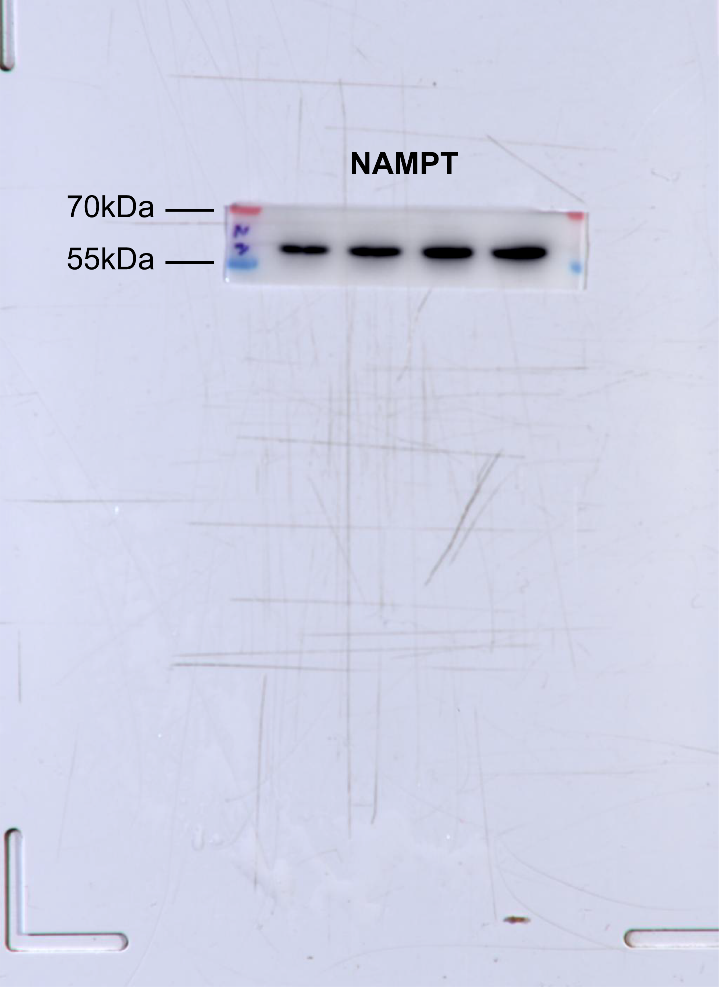

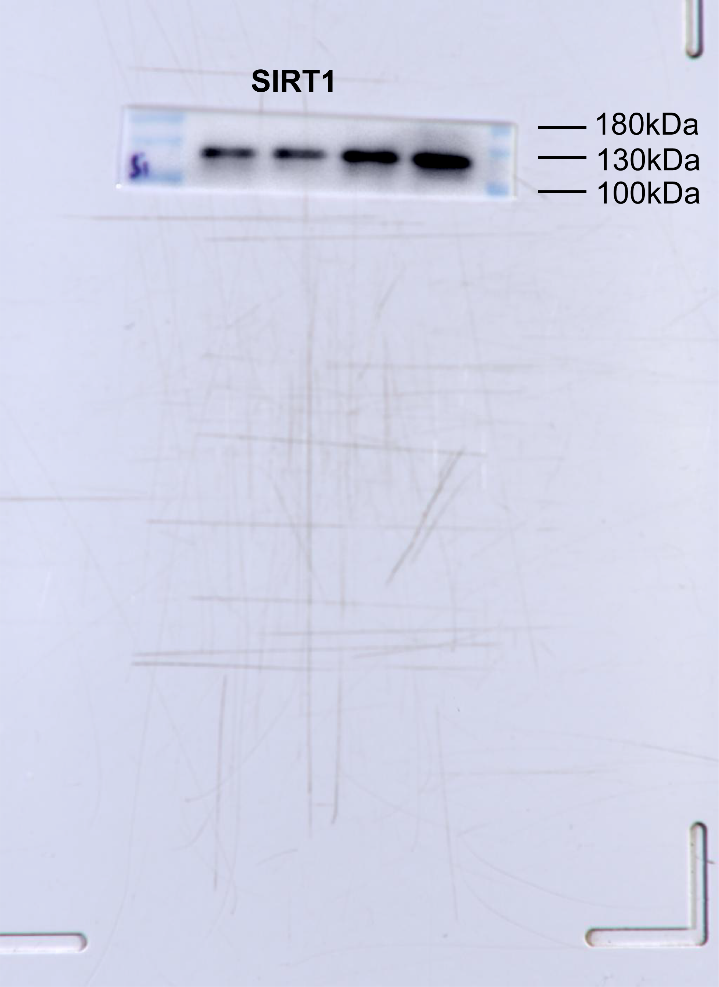

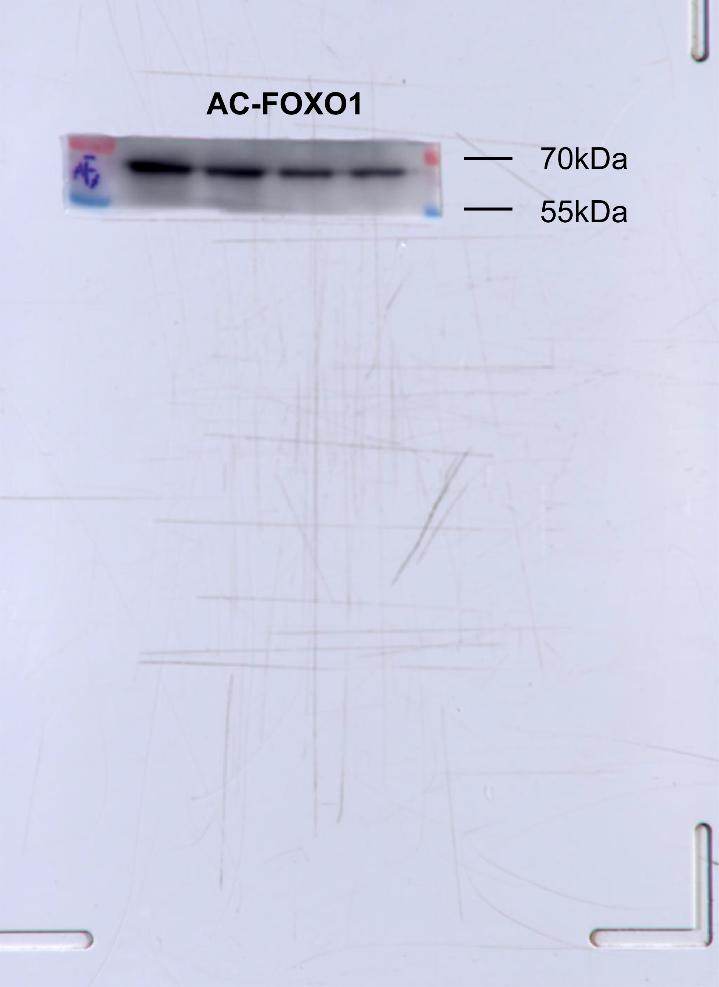

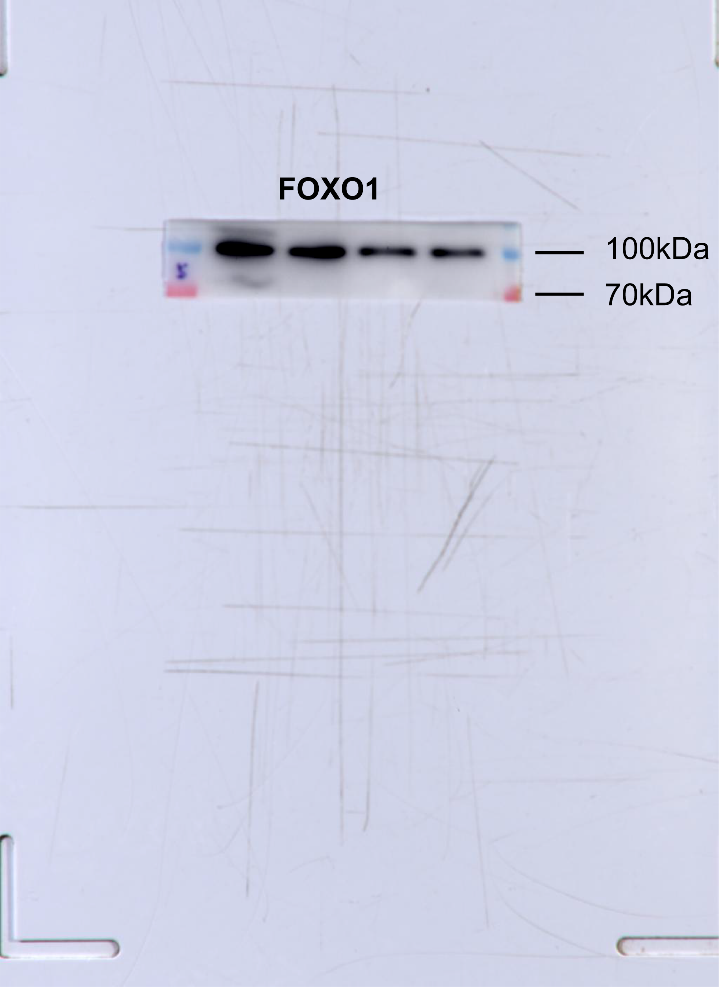

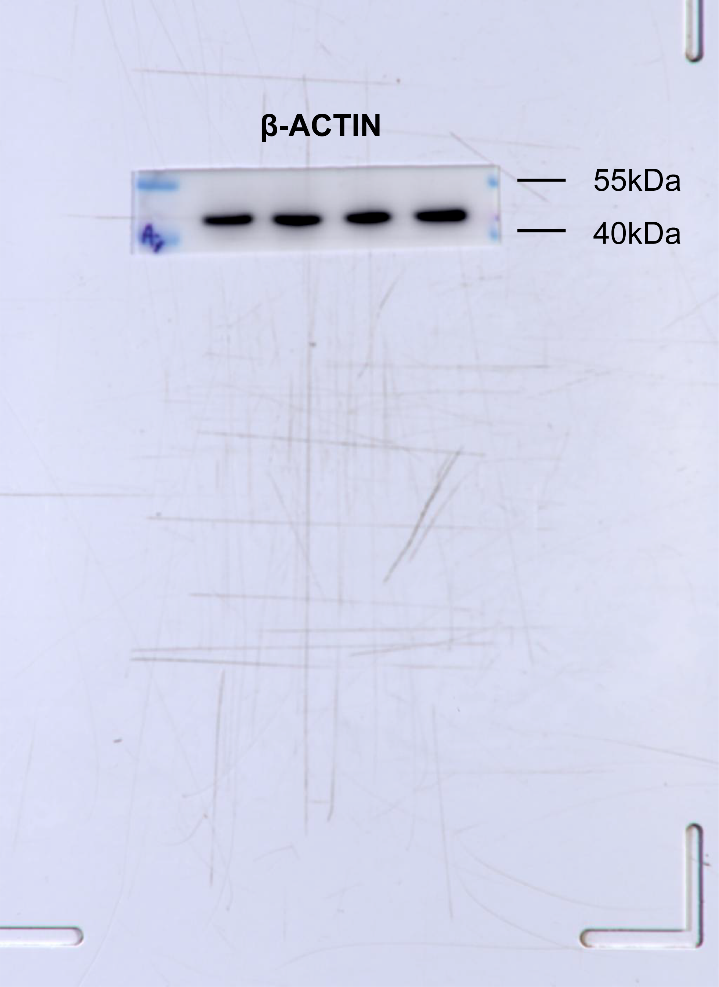


The images of western blotting in Fig 6E.


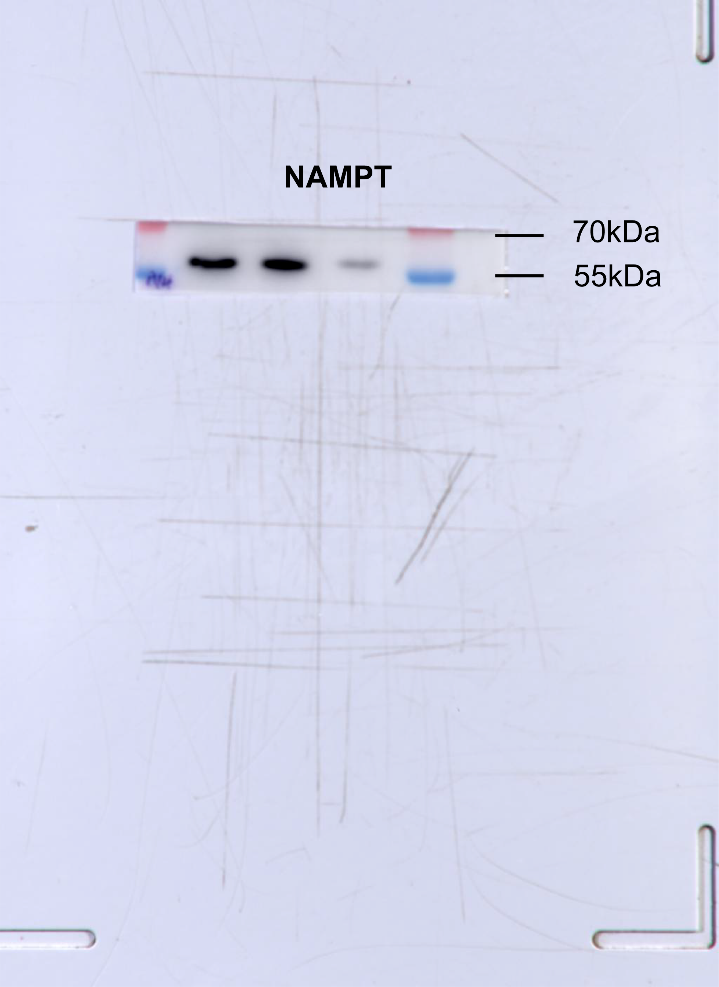

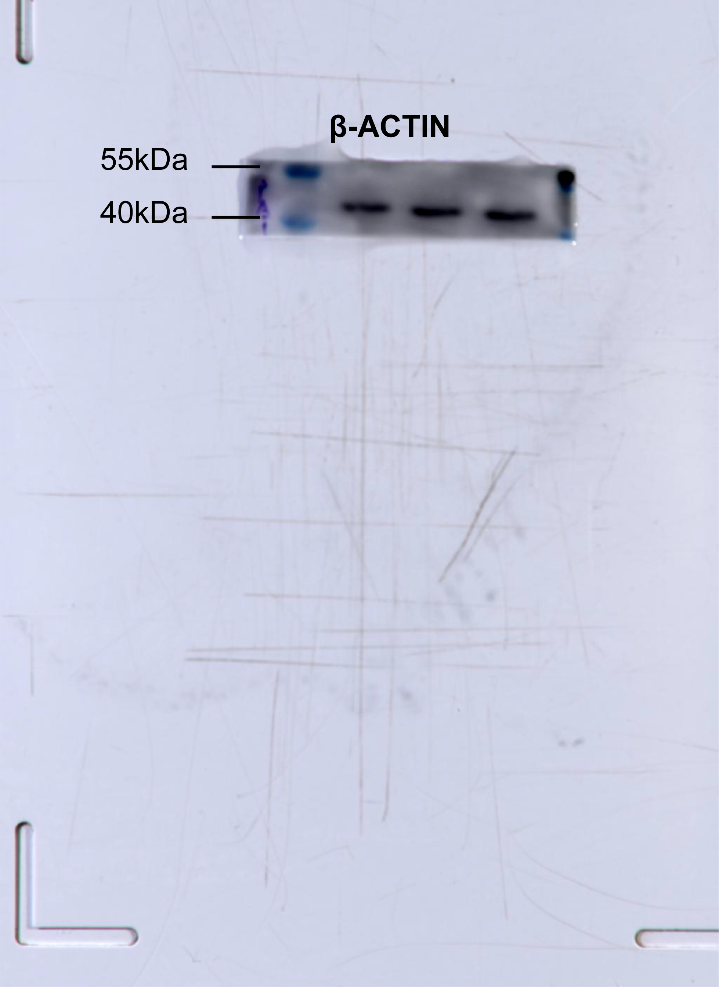


The images of western blotting in Fig 6H.


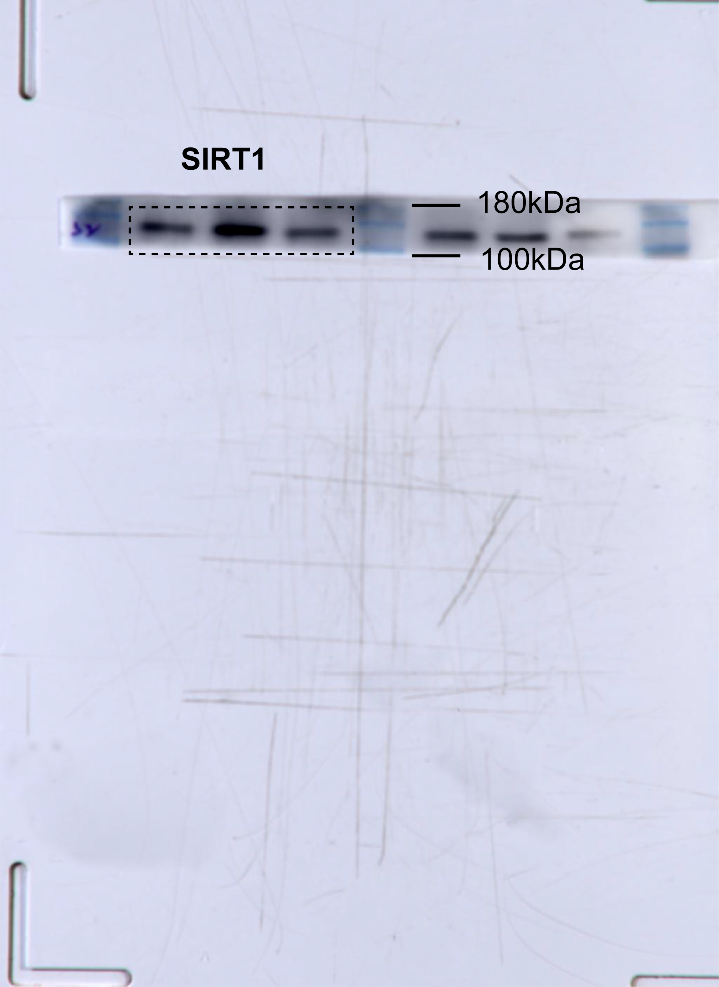

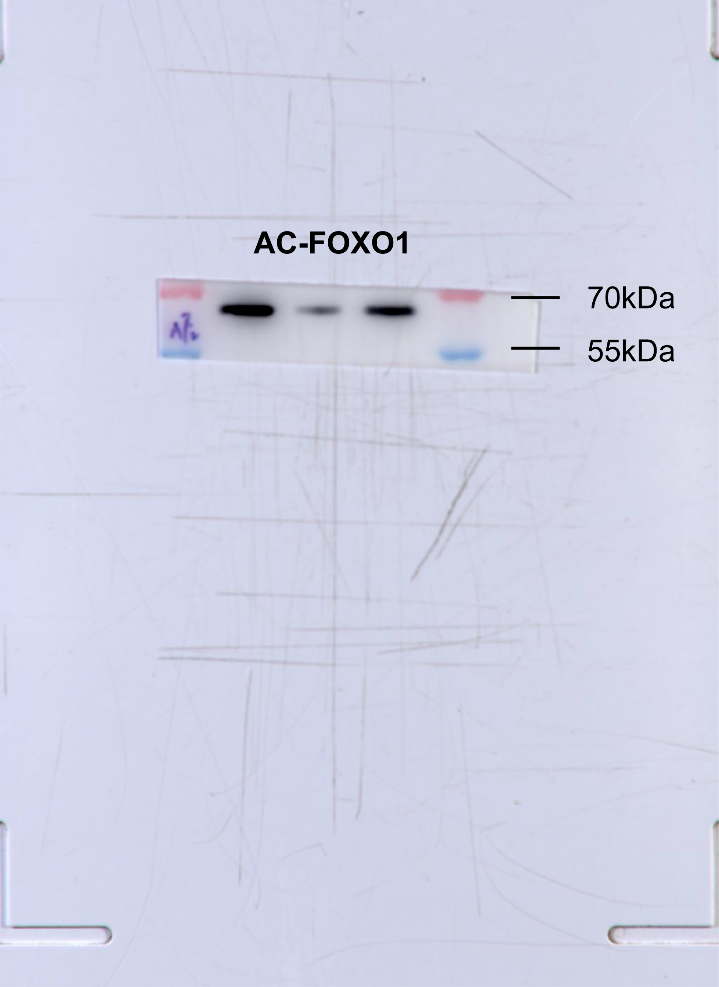

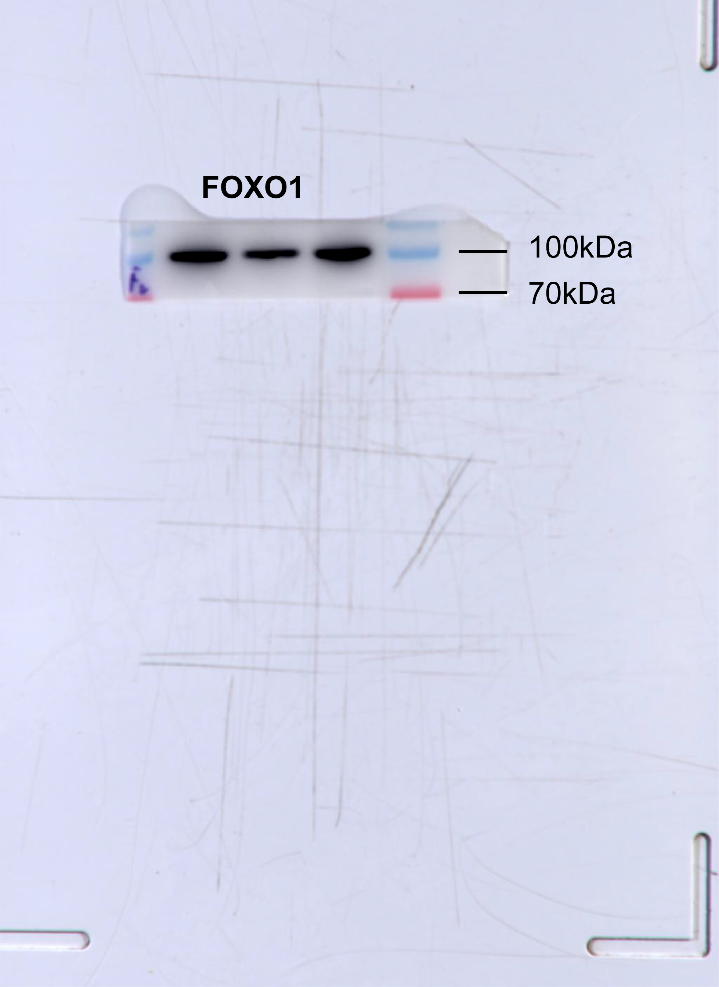

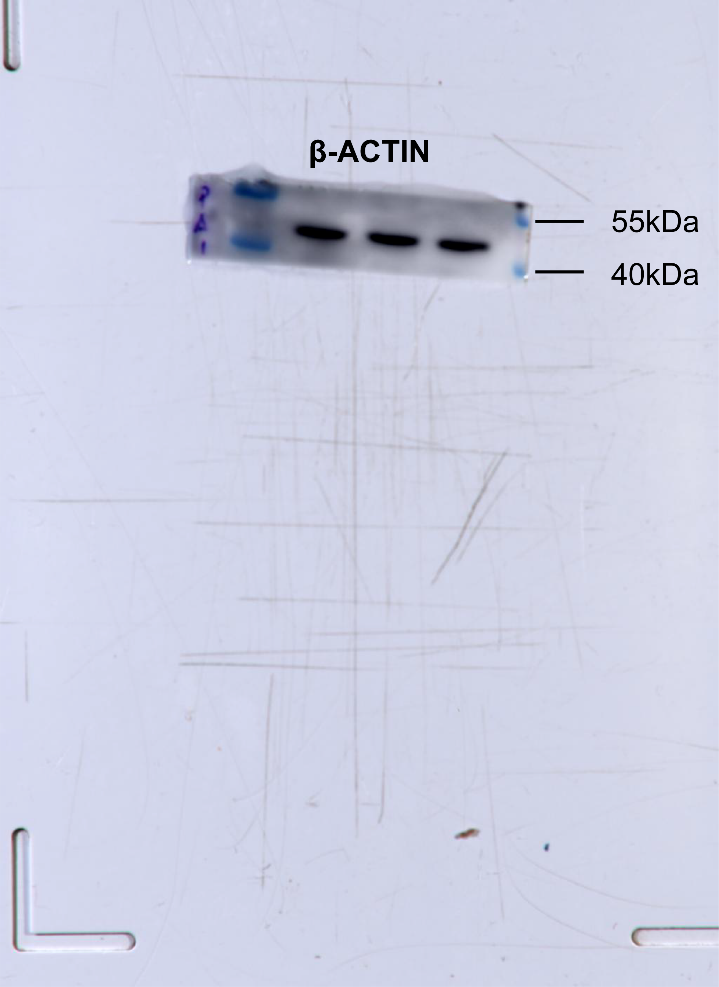


The images of western blotting in Fig 6I.


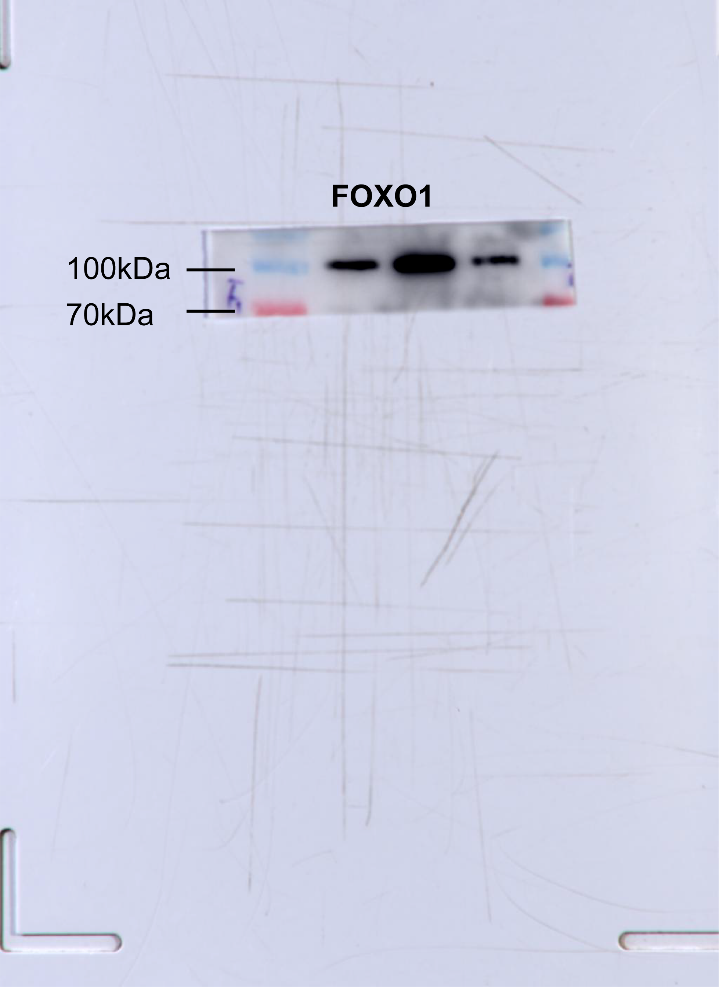

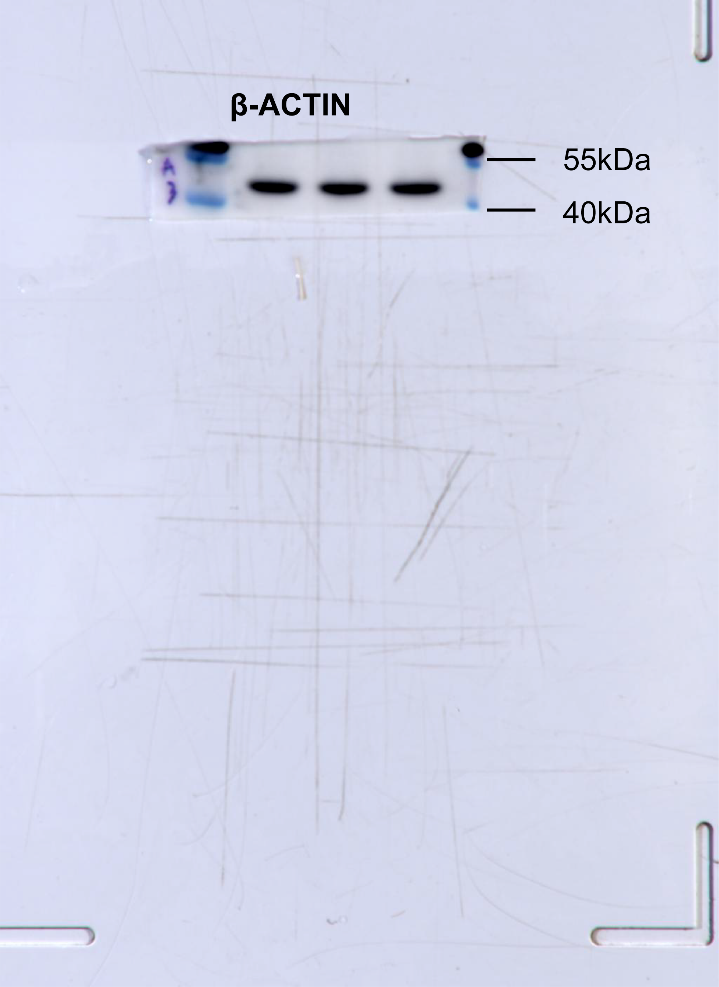

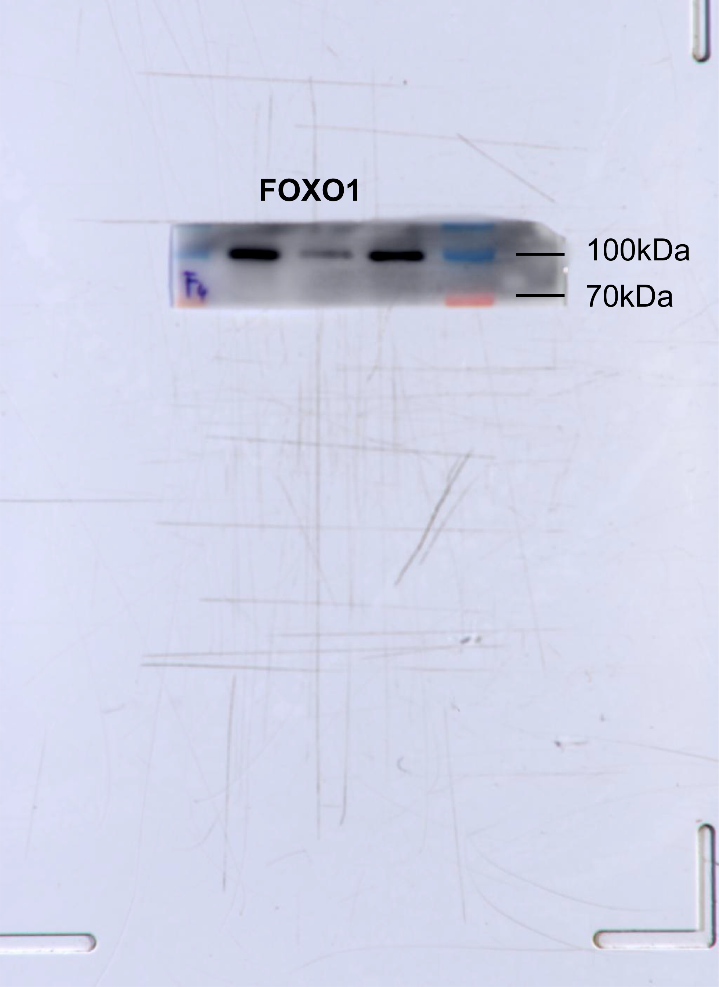

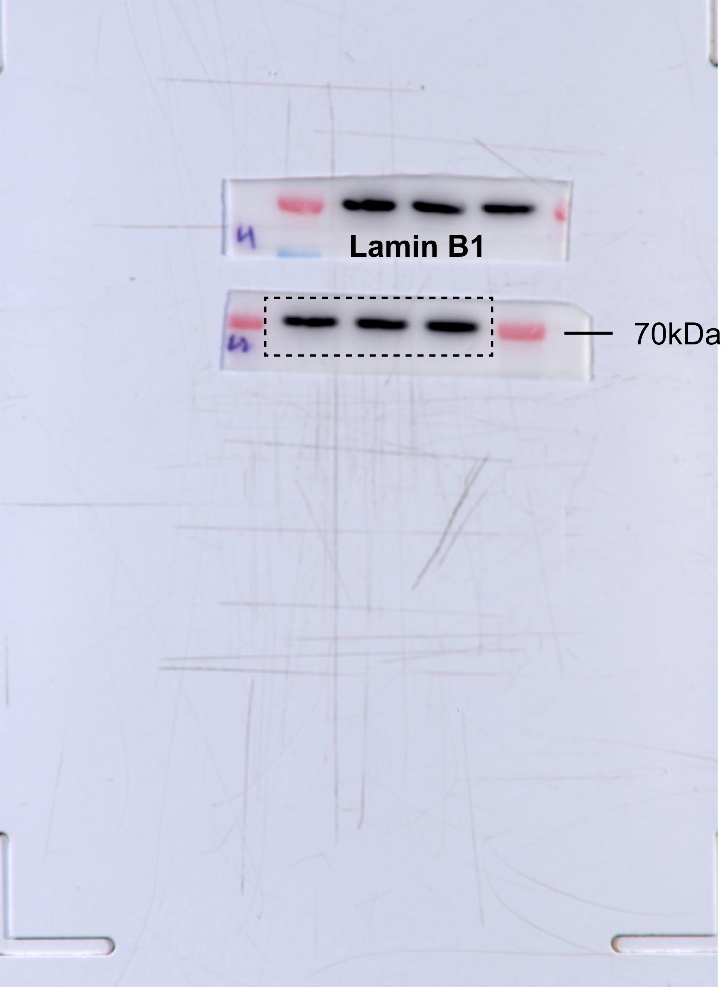


The images of western blotting in Fig 7C.


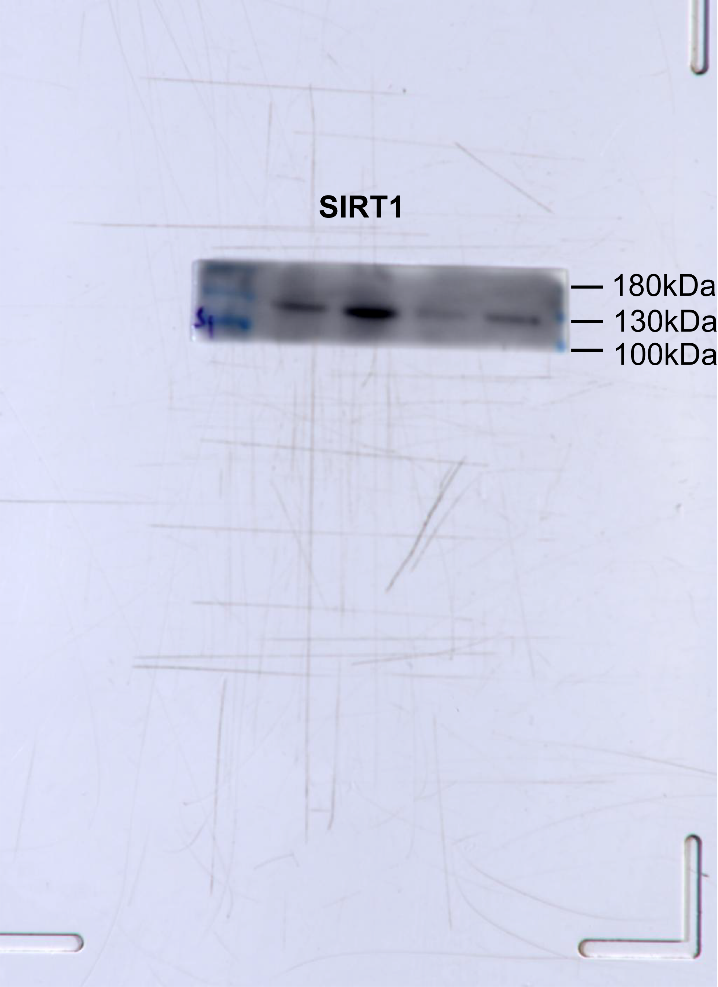

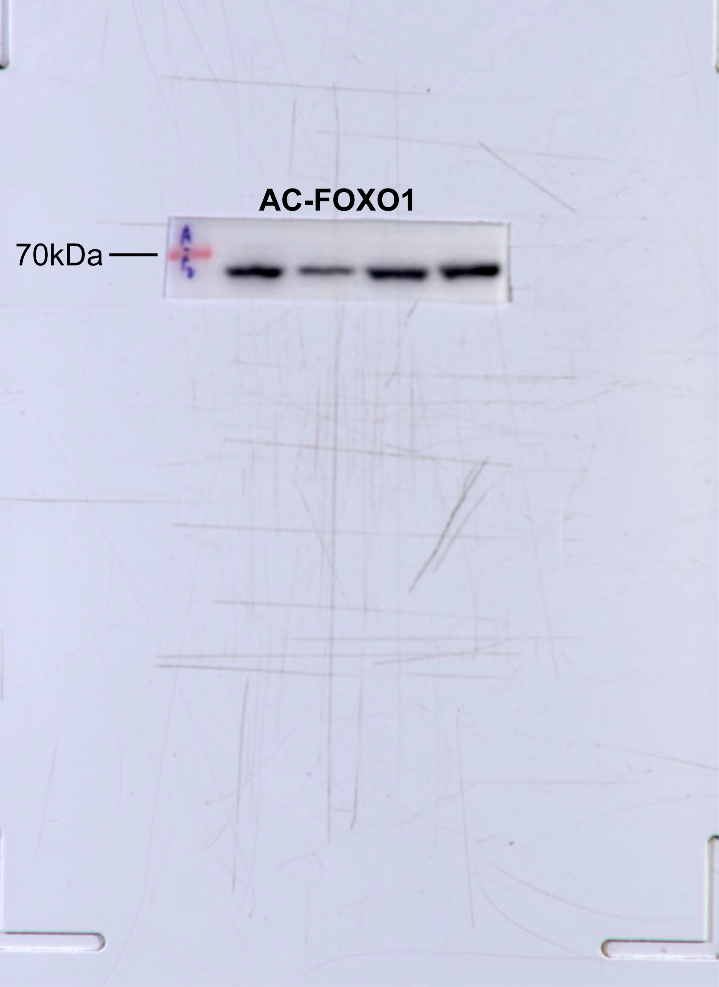

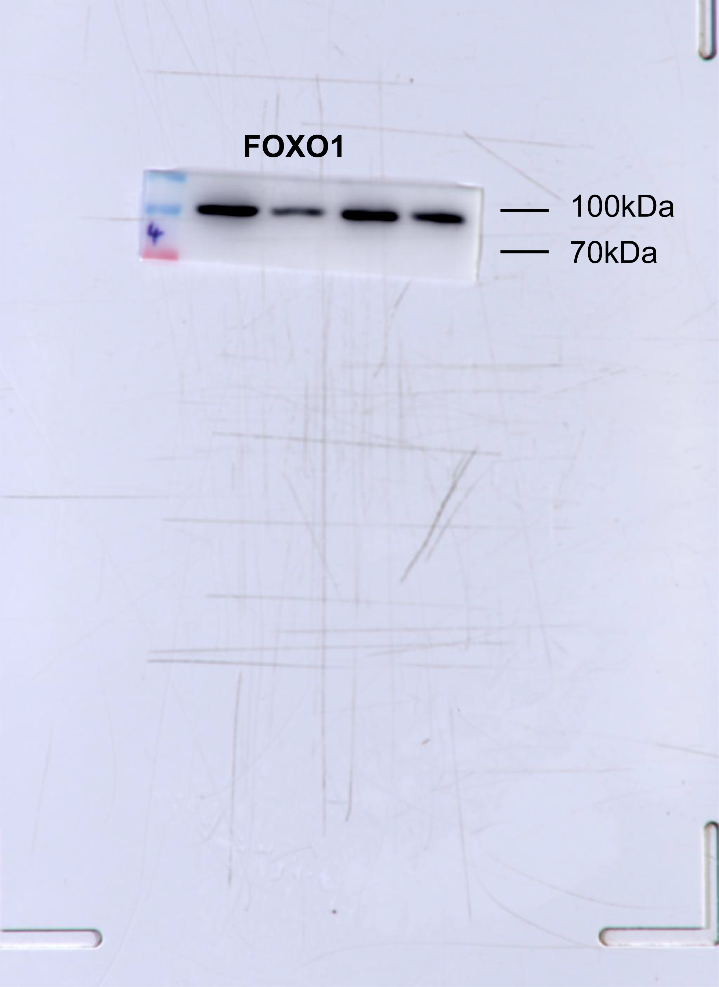

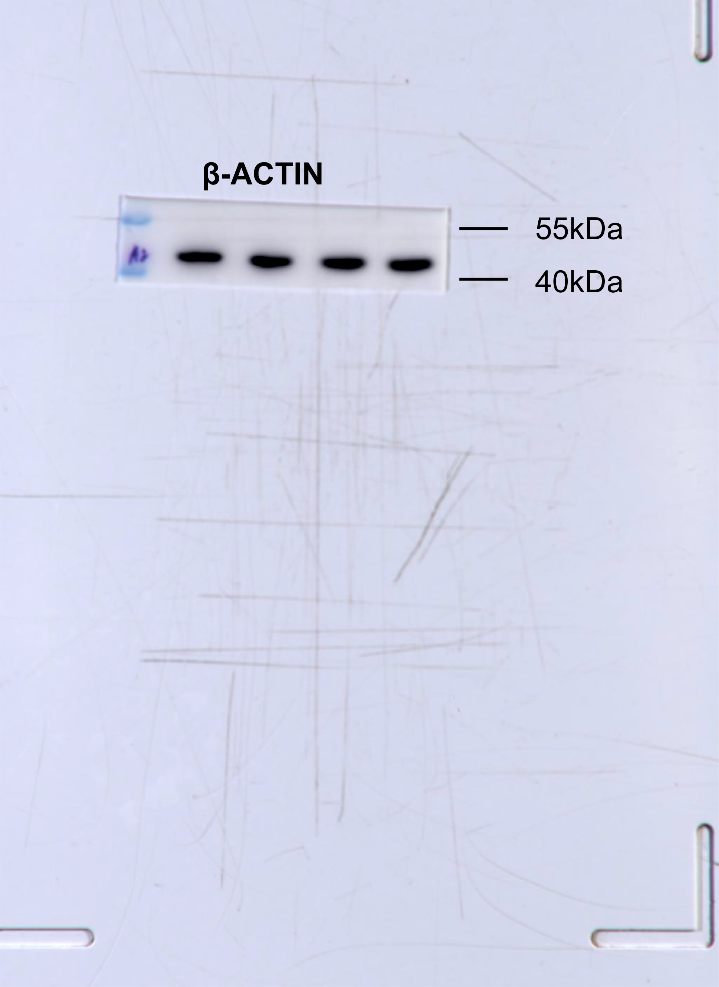


The images of western blotting in Fig 7E.


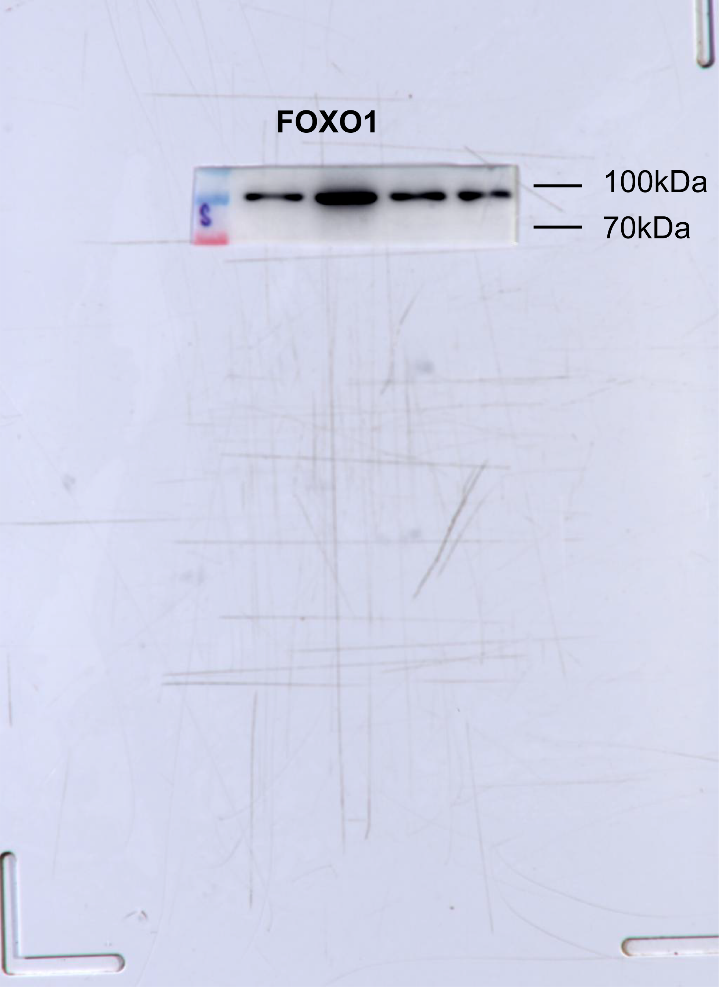

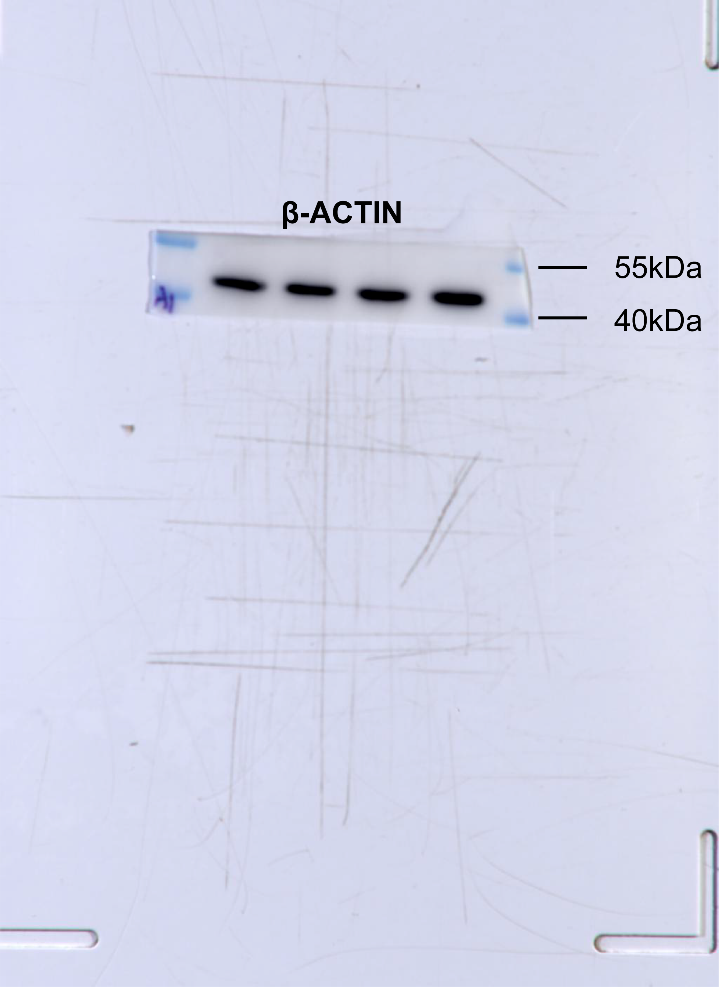

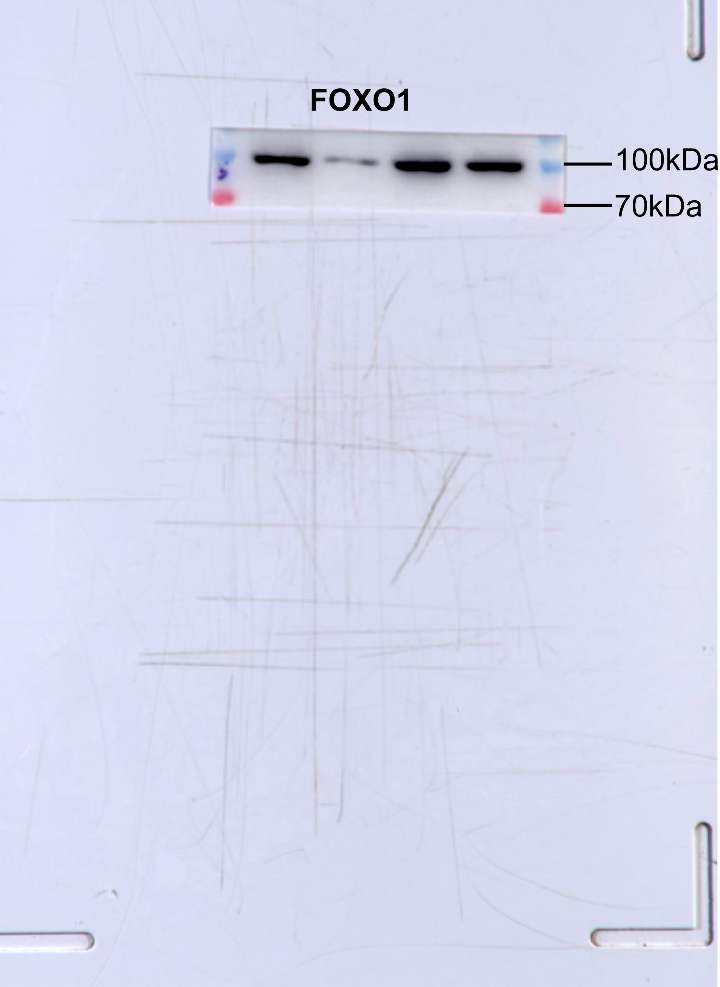

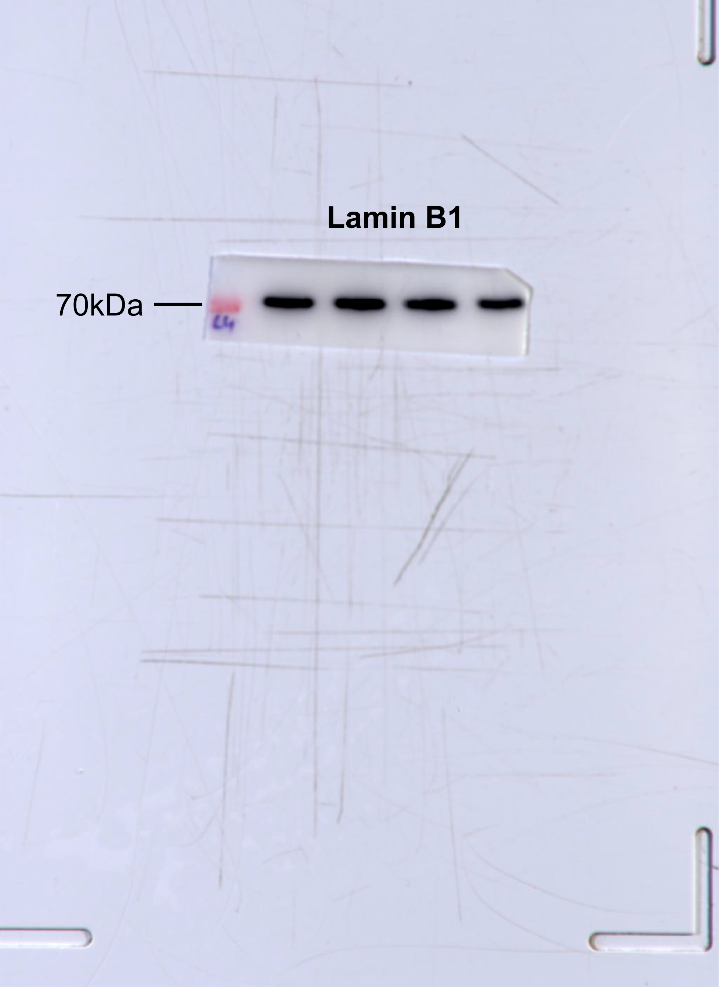

Supplement: Supplementary file 2 — Supplementary Material 2 [file 13287_2025_4245_MOESM2_ESM.docx]
